# NetMiner-an ensemble pipeline for building genome-wide and high-quality gene co-expression network using massive-scale RNA-seq samples

**DOI:** 10.1371/journal.pone.0192613

**Published:** 2018-02-09

**Authors:** Hua Yu, Bingke Jiao, Lu Lu, Pengfei Wang, Shuangcheng Chen, Chengzhi Liang, Wei Liu

**Affiliations:** 1 Nantong Medical College and School of Pharmacy, Nantong University, Nantong, China; 2 State Key Laboratory of Plant Genomics, Institute of Genetic and Developmental Biology, Chinese Academy of Sciences, Beijing, China; 3 University of Chinese Academy of Sciences, Beijing, China; 4 Nantong Polytechnic College, Nantong, China; Kunming University of Science and Technology, CHINA

## Abstract

Accurately reconstructing gene co-expression network is of great importance for uncovering the genetic architecture underlying complex and various phenotypes. The recent availability of high-throughput RNA-seq sequencing has made genome-wide detecting and quantifying of the novel, rare and low-abundance transcripts practical. However, its potential merits in reconstructing gene co-expression network have still not been well explored. Using massive-scale RNA-seq samples, we have designed an ensemble pipeline, called NetMiner, for building genome-scale and high-quality Gene Co-expression Network (GCN) by integrating three frequently used inference algorithms. We constructed a RNA-seq-based GCN in one species of monocot rice. The quality of network obtained by our method was verified and evaluated by the curated gene functional association data sets, which obviously outperformed each single method. In addition, the powerful capability of network for associating genes with functions and agronomic traits was shown by enrichment analysis and case studies. In particular, we demonstrated the potential value of our proposed method to predict the biological roles of unknown protein-coding genes, long non-coding RNA (lncRNA) genes and circular RNA (circRNA) genes. Our results provided a valuable and highly reliable data source to select key candidate genes for subsequent experimental validation. To facilitate identification of novel genes regulating important biological processes and phenotypes in other plants or animals, we have published the source code of NetMiner, making it freely available at https://github.com/czllab/NetMiner.

## Introduction

The complex cellular networks formed by the interacting macro-molecules underlie an organism’s phenotypes [1–3]. Reconstructing a complete map of the cellular networks is crucial for understanding an organism’s genetic architecture underlying complex phenotypes. In animals, multiple types of networks have been constructed based on multi-level ‘-omics’ data sets from genome, transcriptome, proteome, epigenome, metabolome and other subcellular systems [4]. In plants, most of the current available ‘-omics’ data sets come from transcriptome analysis, with relatively few studies generating other types of ‘-omics’ data sets [5]. The rapid accumulation of large-scale and open-access plant transcriptome data derived from the microarray and high-throughput RNA sequencing provides a great opportunity for reconstructing molecular networks underlying diverse biological functions and phenotypes. Co-expression meta-analysis is a classical and powerful method for reconstructing gene functional interaction network using transcriptome data. Based on the hypothesis that the genes with similar expression patterns are often functionally related, this method uses the expression profiles from all available experimental conditions to discover statistically significant functional associations between genes. The extensibility and simplicity make it a powerful tool for inferring the biological roles of uncharacterized genes, understanding the biological processes and gaining novel insight into the global architecture of transcriptome and the molecular mechanism of various phenotypes [5–9].

For co-expression meta-analysis, many algorithms have been proposed to build gene networks. However, it has been shown that the outcome of network inference varies between tools, and the single network inference approach has inherent biases and is unable to perform optimally across all experimental data sets [10,11]. In addition, how to clean-up the links occurring by accident in a gene co-expression network and select the biologically significant associations is also a critical procedure for modeling authentic gene relations [12,13]. Moreover, the current computational methods are mainly designed for analyzing microarray data sets. Indeed, microarrays are intrinsically limited in measuring a small dynamic range of gene expression and only represent a subset of genomic contents (~15000 genes) [8,14]. Compared to microarrays, RNA sequencing (RNA-seq) emerges as a new approach to quantify gene expression in terms of read counts for individual genes, which provides broader dynamic range of measurements allowing whole genome-wide detection of novel, rare and low-abundance transcripts [15]. In RNA-seq, mRNAs are converted to cDNAs, fragmented and sequenced using a high-throughput method to produce short reads. Then these reads are aligned to a reference genome and the expressive abundance of different genomic regions can be computed using the number of mapped reads. By this way, RNA-seq can detect and quantify a large number of novel regions including non-coding genes, such as long non-coding RNA (lncRNA) genes and circular RNA (circRNA) genes, most of which are not to be covered by current microarray platform. However, its potential value in building genome-wide gene co-expression network and predicting the biological functions of novel genes (such as unknown protein-coding genes, lncRNA genes and circRNA genes) has not been well explored. Currently, a great majority of co-expression meta-analyses have neglected the rapid growing availability of RNA-seq samples (especially in the plants). According to our knowledge, only three computational tools tailed for RNA-seq data were developed, including Canonical Correlation Analysis (CCA) [16], SpliceNet [17] and VCNet [18]. These methods reconstructed the high-quality gene co-expression network based on the exon-level, genomic-position-level or allele-level expression information. However, they focused only on evaluating and analyzing the predictive performance of algorithms and several known biological pathways rather than constructing genome-scale co-expression network and predicting the new functions of unknown genes, especially for the non-coding genes. Indeed, these methods are also difficult and even impossible to be applied for building genome-wide gene co-expression network using the large-scale RNA-seq samples (from several hundreds to several thousands) owing to their high computational complexity.

In this study, we have developed a novel ensemble pipeline, called NetMiner, for inferring genome-wide gene co-expression networks using massive-scale RNA-seq samples by integrating the predictions of three different network inference algorithms. We built a network for one species of monocot rice using this method. We compiled a standard physical and non-physical functional gene link data set derived from 4 known biological networks to evaluate the quality of the network using fold enrichment analysis. The quality evaluation was based on the principle that, the larger the ratio of co-expressed genes sharing the same or similar functions, the more valuable the network is. The results showed that our network achieved highest sensitivity and specificity for capturing the functional links between genes when compared with each single method. Moreover, bottom-up subnetwork analysis exhibited the usefulness of our network for solving the practical biological problems. In particular, we demonstrated the potential value of our method for predicting the biological roles of the uncharacterized genome elements, including the protein-coding genes with unknown functions, long non-coding RNA (lncRNA) genes and circular RNA (circRNA) genes. Our study revealed the huge amount of genetic regulatory relationships associated with cellular activities and agronomic traits, which provided a valuable data source for rice genetics research and breeding.

## Materials and methods

### Dataset preprocessing

In this study, we have downloaded 456 primary rice RNA-seq samples from the NCBI Sequence Read Archive (SRA) (see S1 and S2 Datasets for details), with the keywords of “*Oryza sativa*” [Organism] AND “platform illumina” [Properties] AND “strategy rna seq” [Properties] (accessed on May 29, 2014). These RNA-seq samples contain a wide spread of experimental conditions, tissue types and developmental stages. After the SRA files were gathered, the archives were extracted and saved in FASTQ format using the SRA Toolkit. The FASTQ files were first trimmed using Trimmomatic software (version 0.32) [19] with the default settings, except for an additional parameter of minimum read length of at least 70% of the original size. Then, the fastq_quality_filter program included in FASTX Toolkit was adopted to further filtrate the FASTQ files, with the minimum quality score 10 and minimum percent of 50% bases that has a quality score larger than this cutoff value. Surviving RNA-seq samples were mapped to the MSU7.0 reference genomes (55986 genes) using TopHat v2.0.4 with the default settings except for “—max-multihits 1” [20]. The PCR and optical/sequencing-driven duplicate reads were removed using Picard tools. After mapping, the uniquely aligned reads count (RAW) and Fragments Per Kilobase Of Exon Per Million Fragments Mapped (FPKM) of each gene was calculated relative to the reference gene model using the HTSeq-count (v0.5.4) and Cufflinks software (v2.1.1), respectively [21,22]. The unreliable samples and genes were filtered according to the following three criteria: I) The samples, in which the percentage of the number of genes with expression value smaller than 10 reads was larger than 90%, were not considered for further analysis; II) We removed the genes whose expression values were less than 10 reads in more than 80% samples; III) Genes with the variation coefficient of expression values smaller than 0.5 were excluded from subsequent analysis. After filtering, we obtained two expression data sets (RAW reads count and FPKM) composed of 348 RNA-seq samples and 24775 genes. The expression data set of RAW reads count was further normalized using four methods, i.e. I) Upper Quartile (UQ) [23]; II) Trimmed Mean of M values (TMM) [23]; III) Relative Log Expression (RLE) [23] and IV) Variance Stabilizing Transformation (VST) [24]. Consequently, we obtained six RNA-seq gene expression data sets including one RAW reads count data set and five normalized data sets.

The microarray data was extracted from both ATTED-II database and Rice Oligonucleotide Array Database (ROAD) [25,26]. Gene Ontologies (GOs) were downloaded from Plant GeneSet Enrichment Analysis Toolkit (PlantGSEA) [27]. We got the pathway data from two data sources including PlantGSEA and Plant Metabolic Network (PMN) (http://pmn.plantcyc.org/). The transcription factor families were downloaded from Plant Transcription Factor Database (PlantTFDB) [28]. MicroRNAs and their targets were collected from the Plant MicroRNA Target Expression database (PMTED) and Plant MicroRNA database (PMRD) [29]. Known agronomic trait genes were collected from both Q-TARO database [30] and literature. Tos17 mutant phenotypes were extracted from Rice Tos17 Insertion Mutant Database [31]. The phenotypes were associated with MSU 7.0 gene locus identifiers through BLASTN alignments of Tos17 flanking sequences obtained from NCBI website. The protein-protein interaction network of rice was downloaded from PRIN database [32]. Probabilistic functional gene network of rice was obtained from RiceNet data portal [33].

### Gene co-expression network construction

**Fig 1:**
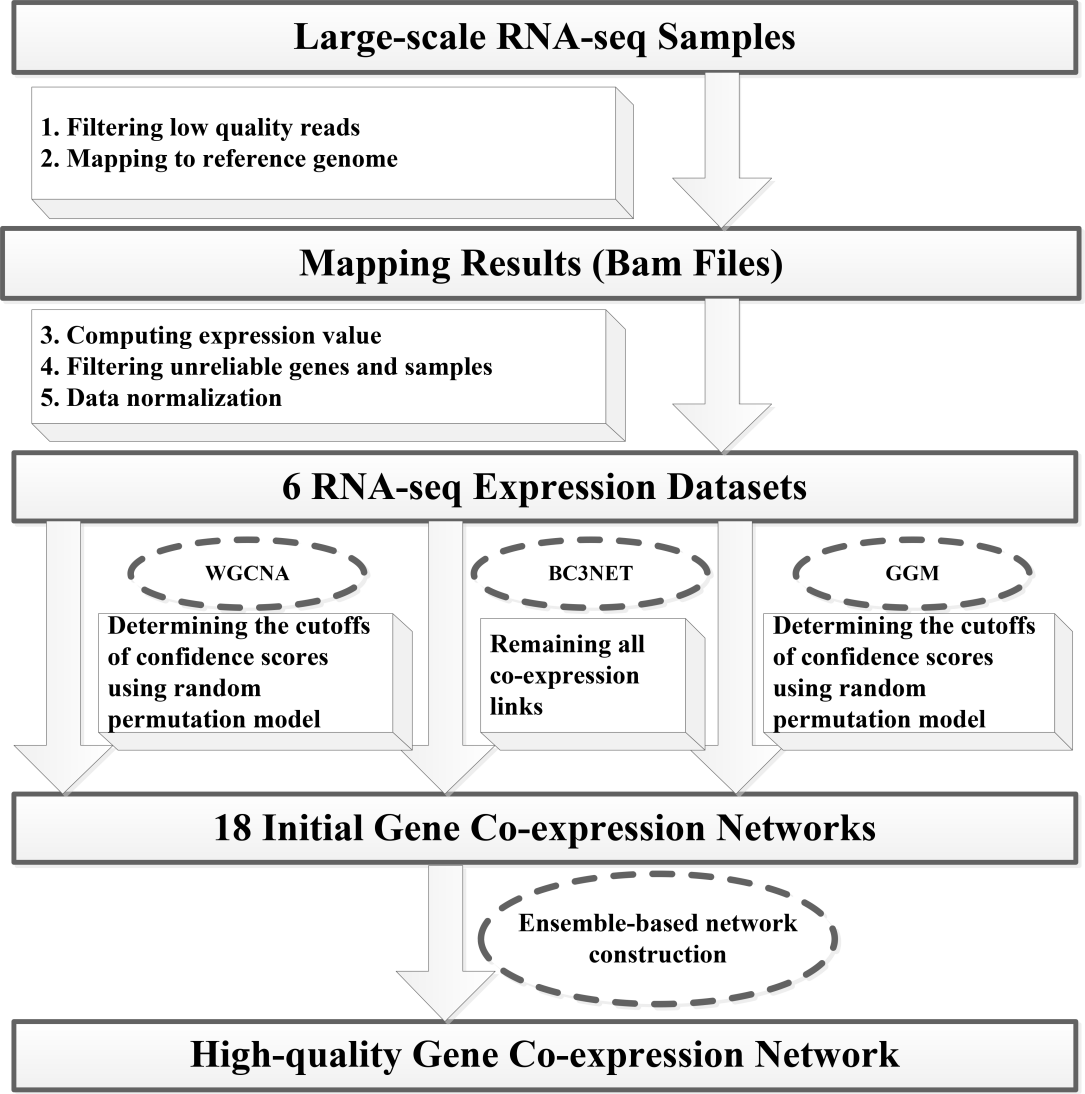
Flowchart of high-quality RNA-seq-based gene co-expression network inference.

We have developed a novel ensemble pipeline for constructing genome-wide and high-quality RNA-seq-based Gene Co-expression Network (GCN) based upon combining multiple inference algorithms, then aggregating their predictions through an unweighted voting system and re-scoring co-expression links. Our ensemble inference system was designed based on the hypothesis that the different network inference methods have complementary advantages and limitations under the different contexts. To select base inference methods for building an ensemble system, five methods were initially tested and evaluated including Graphical Gaussian Model (GGM) [34], Weighted Gene Co-expression Network Analysis (WGCNA) [35], Bagging Conservative Causal Core of Network (BC3NET) [36], Graphical Lasso Model (GLM) [37] and Tree-based Method (TM) [38]. Since GLM and TM have high computational complexity and are unable to be applied for the large number of RNA-seq samples, we have not adopted these two algorithms for subsequent network construction. The flowchart for building RNA-seq-based gene co-expression network was described in Fig 1. In particular, our pipeline was started from short reads filtering and mapping. After reads were filtrated and aligned to reference genome, we computed the gene expression values of each sample, and then removed the unreliable genes and samples. Next, we performed the normalization of expression values and obtained six RNA-seq data sets including RAW reads count data set and five normalized expression data sets. All these steps were described in the Dataset preprocessing section. Based on these 6 RNA-seq data sets, WGCNA, GGM and BC3NET were used to construct 18 initial gene co-expression networks using the R packages of WGCNA, GeneNet and BC3NET, respectively (available from the CRAN repository). Since a great amount of co-expressed gene pairs were outputted from the R packages of WGCNA and GeneNet, we removed those with low confidence scores. For the gene pairs outputted from WGCNA, we used the topological overlap measure as their co-expression confidence score. For gene pairs derived from GeneNet, we used the partial correlation coefficients as their confidence score. We identified a suitable cutoff of confidence score to filter out the low-confidence co-expression links generated by these two methods through a random permutation model. We created 100 random expression data sets by shuffling the associations from genes to expression profiles on the same gene set used by WGCNA and GeneNet and built the randomized gene co-expression networks for each data set. We computed the confidence scores for each random network as background distribution, and used the mean value of 99.99th percentiles of these distributions as the cutoff score to select the high-confidence co-expression links. After obtaining initial networks, we employed a two-step voting procedure to obtain final co-expression network. In the first step. we constructed three consensus gene networks (i.e. intra-method consensus network) by picking the co-expression links included in more than two networks of six initial gene networks. These six networks were built by applying the same inference method (e.g. WGCNA) to six RNA-seq data sets. In the second step, we selected the co-expression links contained in more than one gene network of three intra-method consensus gene networks to establish the final co-expression network. The parameters in two-step voting procedure were decided using the criterion that the reconstructed network is closest to standard positive functional gene links and meanwhile is farthest from standard negative functional gene links.

The calculation procedure of confidence score for each co-expression link of final network was performed as the following: I) Firstly, we normalized the confidence scores of gene co-expression links of each initial network to the interval of range from 0 to 1. II) Then, we assigned a score to each co-expression link of intra-method consensus networks by averaging the normalized confidence scores of all six initial networks. III) Finally, we defined the confidence score for each link of final co-expression network by averaging the confidence scores of three intra-method consensus gene networks. Note that, if a co-expression link did not exist in the network, we assign it a confidence score of 0.

### Performance evaluation

We compiled the real biological data set as replacement a standard set of positive and negative gene functional links to evaluate the quality of network using the following strategy. The gold standard of positive functional links was obtained by capturing the gene pairs that were contained in the same GO categories, in the same pathways, have interacted in the protein-protein interaction network or were linked in the probabilistic functional gene network. To construct the gold standard of negative functional links, we first selected all the biologically unrelated GO pairs (semantic similarity score = 0) that had the number of genes greater than 5 and less than 50, coupling all possible gene pairs of each partnership in these GO pairs as initial non-functional gene relationships. Subsequently, we established 10000 background distributions of gene functional similarity, by 10000 times randomly sampling of 1000 gene pairs and calculating their functional similarities. We have selected a subset of gene pairs from the initial non-functional links as final non-functional links using the criterion that the functional similarity between genes that are smaller than the mean value of 5th percentiles of these simulated background distributions.

Since our real data set included only a subset of true functional and non-functional link space, we evaluated the quality of network using the fold enrichment analysis. The fold of enrichment was calculated as a function of the confidence score cutoff (*k*) in the edge list of the inferred network by the following formula:

$$\frac{n_{k}}{m_{k}}\times \frac{M}{N},$$ (1)

where, *n*_*k*_ is the number of true positive or true negative functional links in the *k*th cutoff of the edge list; *m*_*k*_ is the number of edges of the inferred network in the *k*th cutoff; *M* denotes the number of true positive or true negative functional links in the gold standards and *N* represents the number of all possible interactions in the genome space. The network visualization was carried out using both Cytoscape [39] and BioLayout Express3D [40].

The functional enrichment of co-expression neighborhoods was calculated as the ratio of the relative occurrence in gene set of co-expression neighborhoods to the relative occurrence in genome using Fisher’s exact test. The *p*-value was further adjusted by Benjamini-Hochberg correction for multiple hypotheses testing. The corrected *p*-value smaller than 0.05, was considered as enriched. To evaluate the predictive performance of our RNA-seq-based network for inferring gene function using the co-expression neighborhoods, we adopted the gene-centric evaluation, which was provided in the Critical Assessment of protein Function Annotation (CAFA) project [41]. For this metric, the GO terms of each gene (gold and predicted) were propagated up the GO hierarchy to the root, obtaining a set of terms. In this process, for each scored GO term, we propagated its score (-log(*q*-value) of Fisher’s exact test) toward the root of the ontology with each parent term received the highest score among its children. The Sensitivity (Recall), 1-specificity, Precision and maximum F-measure (F-max) was calculated using the same method as in the CAFA project. The Receiver Operating Characteristics (ROC) curve and Precision-Recall (PR) curve was drawn by changing the threshold and plotting the Sensitivity versus the 1-specificity. Similarly, we obtained the Precision-Recall (PR) curve by altering the threshold and plotting the Precision versus the Recall. Semantic similarities between the GO term pairs were calculated using GOSim [42]. Functional similarities between genes in terms of the GO space were calculated using the metric adopted from one reference paper [43].

### Analysis of circRNA genes

The circular RNA (circRNA) genes were predicted using 618 novel rice RNA-seq samples downloaded from the NCBI Sequence Read Archive (accessed on February 15, 2016) by CIRI software [44]. We have calculated the counts of junction reads of a circRNA as its relative expression abundance. Then, we integrated the aligned reads number of known rice genes using HTSeq-count program (v0.5.4) and expression values of circRNAs into a numeric expression matrix. We removed the circRNAs from the matrix if it was identified in less than 3 RNA-seq samples. Using the filtered matrix, we built three initial gene co-expression networks by WGCNA, GGM and BC3NET. Based on this, we have selected the co-expression links contained in more than one network of the three initial networks to obtain the final co-expression network. Although only the numbers of junction reads were adopted to measure the expression abundances of circRNAs, this method is simple and effective for building gene co-expression networks, given that the reads were distributed uniformly along circRNA.

## Results and discussion

### Network construction and evaluation

To evaluate the quality and reliability of downloaded RNA-seq samples, we have analyzed 348 rice RNA-seq transcriptomes after removing the unreliable genes and samples (S1 and S2 Datasets, see Materials and methods for details). After reads filtering and trimming, a total of 12,458,505,209 reads remained in the samples, 75% of which were mapped to the rice MSU7.0 reference genome and 71% of which were mapped uniquely (S2 Dataset). Of the genes covered with RNA-seq reads, 98% have coverage of > 50% of gene length (Figure A in S1 Fig). Although there exist very large differences in the number of mapped reads between samples, the percentage of expressed genes was similar in most of them, ranging from 32% (10th percentile) to 66% (90th percentile), and as the number of mapped reads increased, the proportion of the number of expressed genes rapidly increased to saturation (see Figure B in S1 Fig). We have tested several normalization methods to compute the expression abundance and expression correlations between genes and samples. The tissue-specific expression patterns and enrichment results of rice genes suggested that these RNA-seq samples were highly reliable (see S1 Text, S2–S6 Figs,—S1 Table and S3 Dataset for details).

We comprehensively analyzed whether the co-expression between genes is to be associated with their biological functions. Our results demonstrated that the functionally related genes are often to be co-expressed in these rice RNA-seq samples (see S1 Text, S7 and S8 Figs and S4 Dataset for details). This indicated that the co-expression links inferred by RNA-seq data can be adopted to predict gene functions. To find the novel functions of unknown genes, we designed a new ensemble pipeline to construct high-quality RNA-seq-based gene co-expression network based upon an un-weighted voting system and rescoring the gene co-expression links. This pipeline combines 18 initial networks inferred by three state-of-the-art inference algorithms, including Graphical Gaussian Model (GGM) [34], Weighted Gene Co-expression Network Analysis (WGCNA) [35], Bagging Conservative Causal Core of Network (BC3NET) [36], learning on 6 different types of RNA-seq expression data sets (see Materials and methods for details). We have selected these three inference methods but not the other existing approaches [16–18,37,38] due to either the high computational complexity or the inconsistent data source (Table 1).

**Table 1:** A table summing up the main features of different algorithms.

Feature	NetMiner	WGCNA	GGM	BC3NET	GLM	TM	ARACNE	VCNet	CCA	SpliceNet
Computational complexity	Middle	Low	Low	Middle	High	High	Low	High	High	High
Whole genome inference	Yes	Yes	Yes	Yes	Yes	Yes	Yes	No	No	No
Data level	Gene	Gene	Gene	Gene	Gene	Gene	Gene	Exon	Exon/Position/Allele	Exon
Sample size	Large-scale	Large-scale	Large-scale	Large-scale	Large-scale	Large-scale	Large-scale	Small-scale to middle-scale	Small-scale to middle-scale	Small-scale to middle-scale

We have constructed the co-expression network of rice, which includes 16770 genes with 146,419 links. We then ranked these co-expression links according to their confidence scores in a descending order (see Material and methods for details). This network showed the small-world characteristic with an average path length between any two nodes equal to 6.28. The distribution of node degrees obeyed the truncated power-law where most nodes have a few co-expression partners with only a small ratio of nodes associating with many partners (Figure A in S9 Fig). The negative correlation between node degrees and clustering coefficients revealed the hierarchical and modular characteristics of network and synergistic regulation of gene expression (see Figure B in S9 Fig) [7].

**Fig 2:**
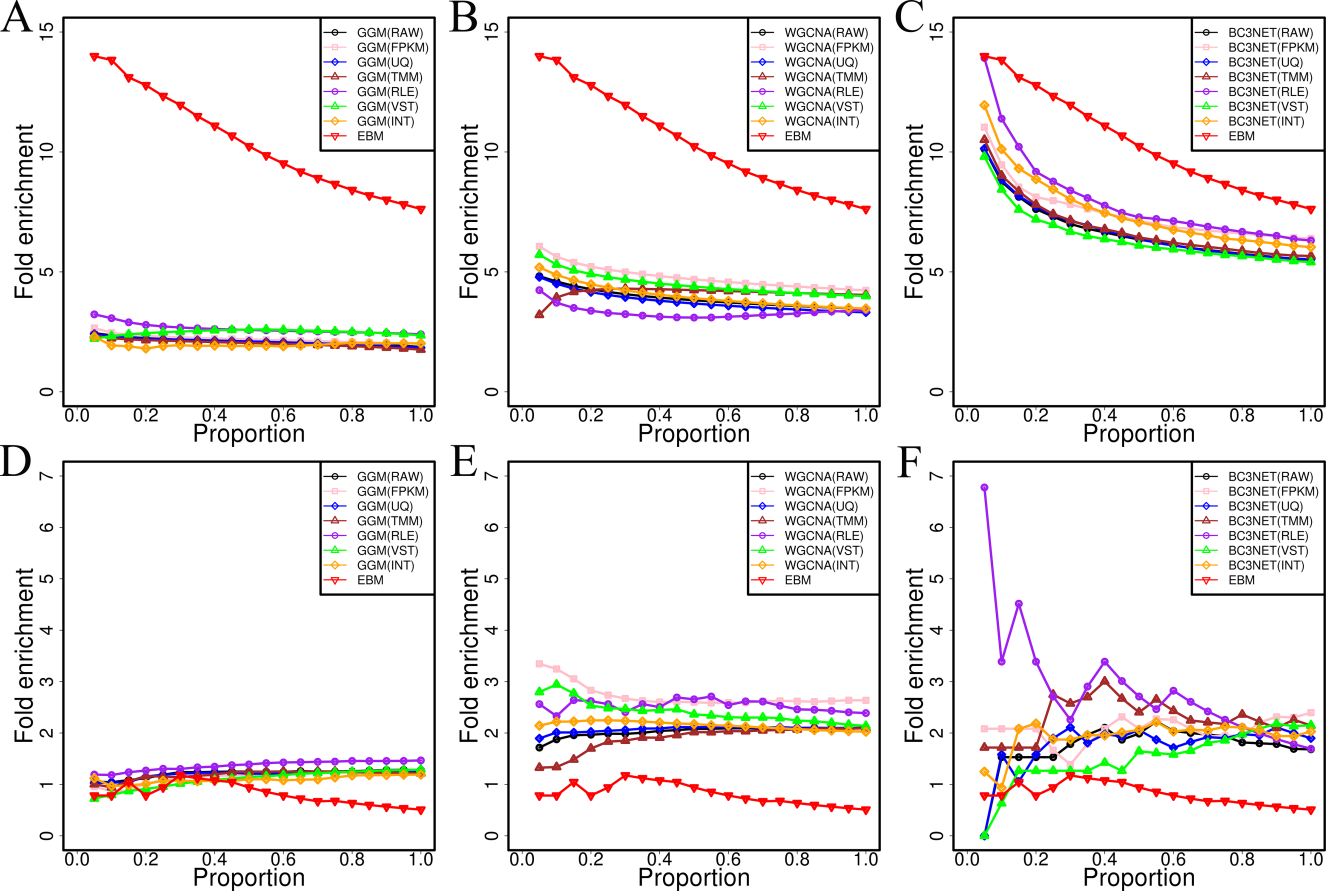
Enrichment folds of different algorithms for co-expression network inference. A) Comparing to GGM for standard positive links. B) Comparing to WGCNA for standard positive links. C) Comparing with BC3NET for standard positive links. D) Comparing with GGM for standard negative links. E) Comparing with WGCNA for standard negative links. F) Comparing with BC3NET for standard negative links. In the legends, the RAW, FPKM, UQ, TMM, RLE and VST represented the networks obtained by the single RNA-seq data set; INT indicated intra-method consensus networks established by integrating the predictions of different RNA-seq data sets, EBM denoted high-quality gene co-expression network obtained by integrating all intra-method consensus networks.

We assessed the quality of this network based on the principle that, the more co-expression relationships connect the genes sharing similar biological functions, the more reliable the network was. For evaluation, we compiled a standard set of positive gene functional links (9390203 interactions), by capturing gene pairs that were contained in the same functional categories or were connected in known biological networks, and a standard set of negative gene functional links (272997 interactions) based on the functional dissimilarities between genes (see Materials and methods for details). We used fold enrichment analysis to measure the relationships between our network and these two standard functional links: The larger the proportion of the number of shared elements divided by that expected by random chance, the closer they were (for details, see Materials and methods). We first analyzed the closeness between our gene network and standard positive functional gene links to evaluate the sensitivity of our network. Then, we further analyzed the distinctiveness between our gene network and standard negative functional gene links to assess the specificity of our network. We found that the network structure obtained by our ensemble inference method was consistently better than the networks built by the individual method with higher enrichment level for standard positive links and lower enrichment level for standard negative links (Fig 2). These results suggested that the committee of different methods can reduce the bias occurring in a single inference method and provide more reliable predictions with higher sensitivity and specificity. We have observed that the enrichment levels of the integrated gene network built using six RNA-seq data sets have not obviously changed (such as GGM method, Fig 2A, line highlighted in yellow) than the initial network using each single data set, indicating that integrating the gene networks built using different types of RNA-seq data sets had no obvious effects on the structure of inferred gene network. Besides, this might also indicate that each of six gene networks derived by single data set was focused on different partitions of cellular transcriptome (Giorgi et al., 2013).

We tested whether the network constructed by the weighted voting method [10] was better than which built by our un-weighted voting method. We obtained the weighted network by scoring each single inference method’s inference performance. The performance weight value of a single inference method was obtained by dividing the enrichment fold of its resulting network on standard positive links by which on standard negative links. We adopted the paired *t*-test to assess the performance differences between two networks using the values of standard link enrichments in 20 different cutoff scores of co-expressions. Though the weighted voting improved the performance when compared with un-weighted voting, the difference was not statistically significant (*p*-values = 0.20 and 0.16 for standard positive and negative links, respectively). This is consistent with Marbach et al. study [10], which claimed that integrating all inference methods using unweighted voting seems to be a good choice since the performance of an inference method was difficult to estimate when inferring an unknown gene network. We also examined whether the edge confidence (rank) average method [10] were more effective than un-weighted voting method in building gene co-expression network. To compare with these two methods, we first normalized the confidence scores (ranks) of initial gene co-expression links inferred from each data source and algorithm to an interval range from 0 to 1. For each co-expression link, we then assigned the mean value of confidence scores (ranks) in initial networks as its probability score in the final network. We found that edge confidence average method had not improved the enrichment level of positive standard links but it increased the enrichment level of negative standard links when compared to our unweighted voting method. For edge confidence average method, the *p*-values were 4.64E-3 and 5.67E-3 for standard positive and negative links, respectively. For edge rank average method, the *p*-values were 2.44E-4 and 1.81E-3 for standard positive and negative links, respectively. Using the similar method, we next evaluated whether our proposed method has better performance than a classic gene network inference algorithm, ARACNE [45]. We applied this method to six RNA-seq expression data sets to obtain six gene networks, and then compared them with our gene network. The results showed that, for standard negative links, the networks built by ARACNE had higher enrichment than our network, while its enrichment level was lower than our network for the standard positive links (The mean *p*-values equaled 2.64E-09 and 3.61E-08 for the standard positive and negative links, respectively). This result indicated that our method can more accurately reconstruct gene co-expression network than ARACNE method. As reported in previous studies, three powerful methods, CCA, SpliceNet and VCNet, used the expression information of sub-gene-level (e.g. exon-level) to detect co-expressions between genes. However, these methods were unable to be applied to construct genome-wide gene co-expression network using the large number of RNA-seq samples in an acceptable computation cost.

We further analyzed the effects of expression sample numbers and expression abundances of co-expression links on the enrichment level of standard links. The expression sample number of a co-expression link, connecting two genes A and B, was defined as the total number of samples which plus the number of gene A expressed samples and the number of gene B expressed samples. The expression abundance of a co-expression link, connecting two genes A and B, was defined as the expression abundance summation of gene A and gene B in all samples. Interestingly, we have found that the co-expression relationships connecting highly or frequently expressed gene pairs were positively associated with the standard positive links and were negatively associated with the standard negative links (see S10 Fig). We also observed that the expression sample number of co-expression link is a more reliable factor than the expression abundance to affect the enrichment level of standard links (S10 Fig). Subsequently, we examined whether a large fraction of gene interactions was recovered by our collected rice RNA-seq data. The results demonstrated that most of the general transcriptional links were already mined reliably with 348 rice RNA-seq samples (see S2 Text for details).

### Prediction of gene functions through co-expression subnetworks

We observed that our reconstructed RNA-seq-based gene co-expression network was always a positive predictor for the protein-protein interaction network, probabilistic functional gene network, GO network and pathway network (see S3 Text and S11 Fig for details). Meanwhile, we also found that many genes under the same GO categories were more connected to each other than expected by chance (see S4 Text and S5 Dataset for details). Therefore, we adopted GO enrichment analysis of a gene’s co-expression neighborhood as a tool to predict its biological functions [46]. For each gene belonging to a given GO category, we asked whether GO enrichment in its co-expression neighborhoods could predict its correct function. An inference was called true positive, if and only if the predicted GO term was more specific than its known GO terms or was equal to known GO terms. In an enrichment significance level of the corrected *p*-value smaller than 0.05, we found that 15.50% (Sensitivity) of annotated functions were correctly inferred based on 10545 annotated genes in rice network. If we used only gene annotations on the second and third layers of the directed GO graph for inference, the Sensitivity was increased to 21.66%. We found that 21.27% (Precision) of all inferred functions were true positives and this number was improved to 25.38% when we adopted the second and third layers of directed GO graph. These results might be suggesting the incompleteness or errors in the GO annotations of rice genes.

**Fig 3:**
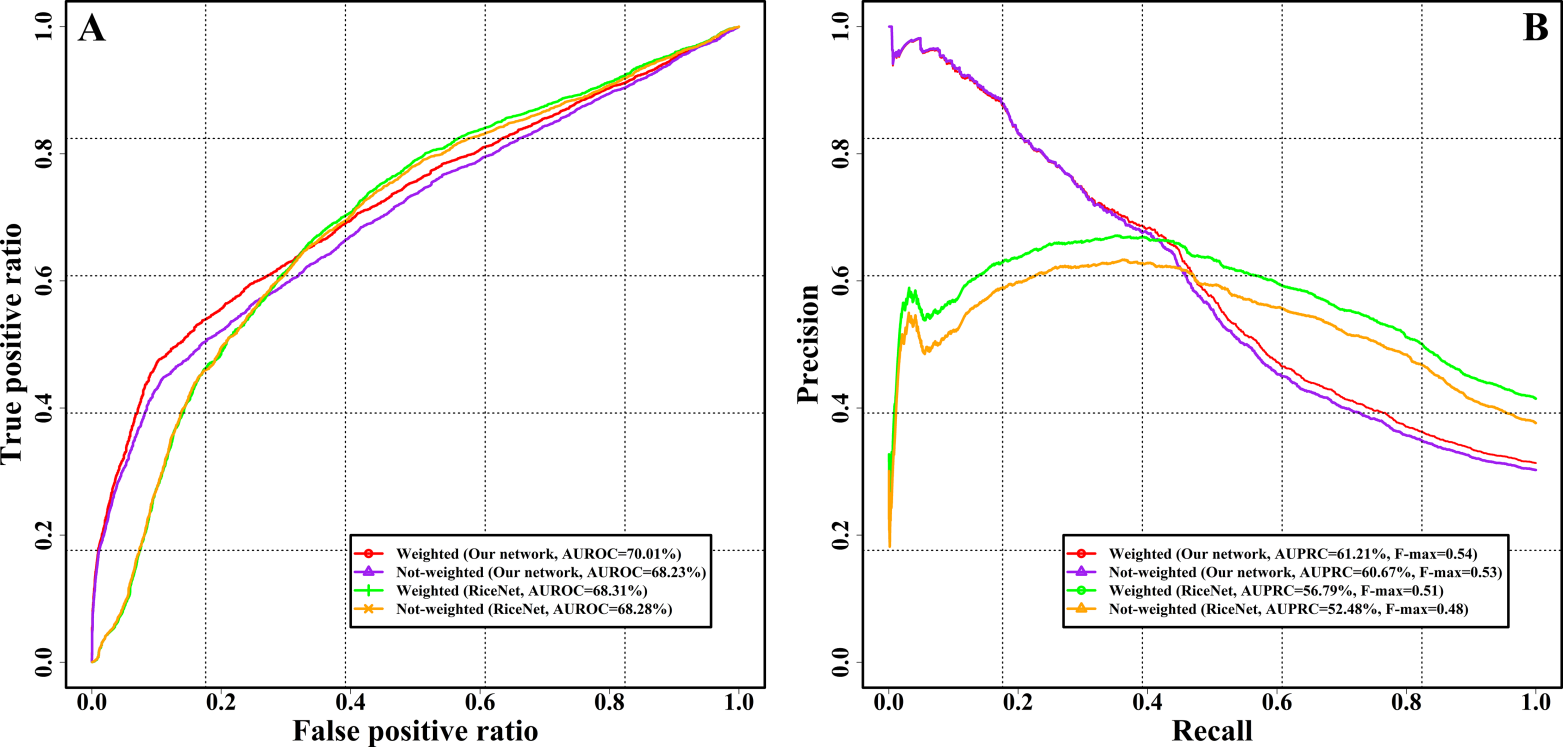
Performance evaluation of our network for predicting gene function. A) Receiver Operating Characteristics (ROC) curve. B) Precision-Recall (PR) curve. In the legends, Not-weighted indicated that the evaluation parameters were calculated by the standard method of CAFA project; Weighted indicated that the evaluation parameters were calculated by the weighted method of CAFA project.

The relatively low Sensitivity and Precision of our network in inferring gene function might be due to simple scoring metrics. We further analyzed the predictive performance of our network based on the Critical Assessment of protein Function Annotation (CAFA) metrics [41] (see Materials and methods for details). To eliminate the effects of the incompleteness and errors of GO annotations, we removed the genes with I) the number of known annotations smaller than 3; II) the number of predicted annotations smaller than 3 and III) the variation coefficient of the number of known annotations and the number of predicted annotations larger than 0.5. We calculated Sensitivities, 1-specificities and Precisions under different significance thresholds (-log(corrected *q*-value)) to produce the Receiver Operating Characteristics (ROC) and Precision-Recall (PR) curves. For correcting the different depths of GO predictions, we further calculated the weight value of each GO term and plotted the weighted ROC and PR curves. The weighted ROC and PR curves got the larger AUROC score (70.01%), AUPRC score (61.21%) and maximum F-measure (F-max = 0.54) than not-weighted one (AUROC = 68.23%, AUPRC = 60.67% and F-max = 0.53) (see Fig 3), indicating that our network could effectively predict the difficult or less frequent GO terms. In addition, we also compared the predictive performance of our gene network with RiceNet using the same evaluation criteria as employed in our study. We observed that our co-expression network was comparable or better than the RiceNet in terms of the ROC and PR curves (Fig 3). Moreover, we also found that the semantic similarities between the known GO terms and our predicted GO terms were obviously higher than random control terms (*p*-value = 5.24E-10, paired *t*-test). These results indicate that our gene network can be effectively applied for inferring the potential functions of unknown genes.

**Fig 4:**
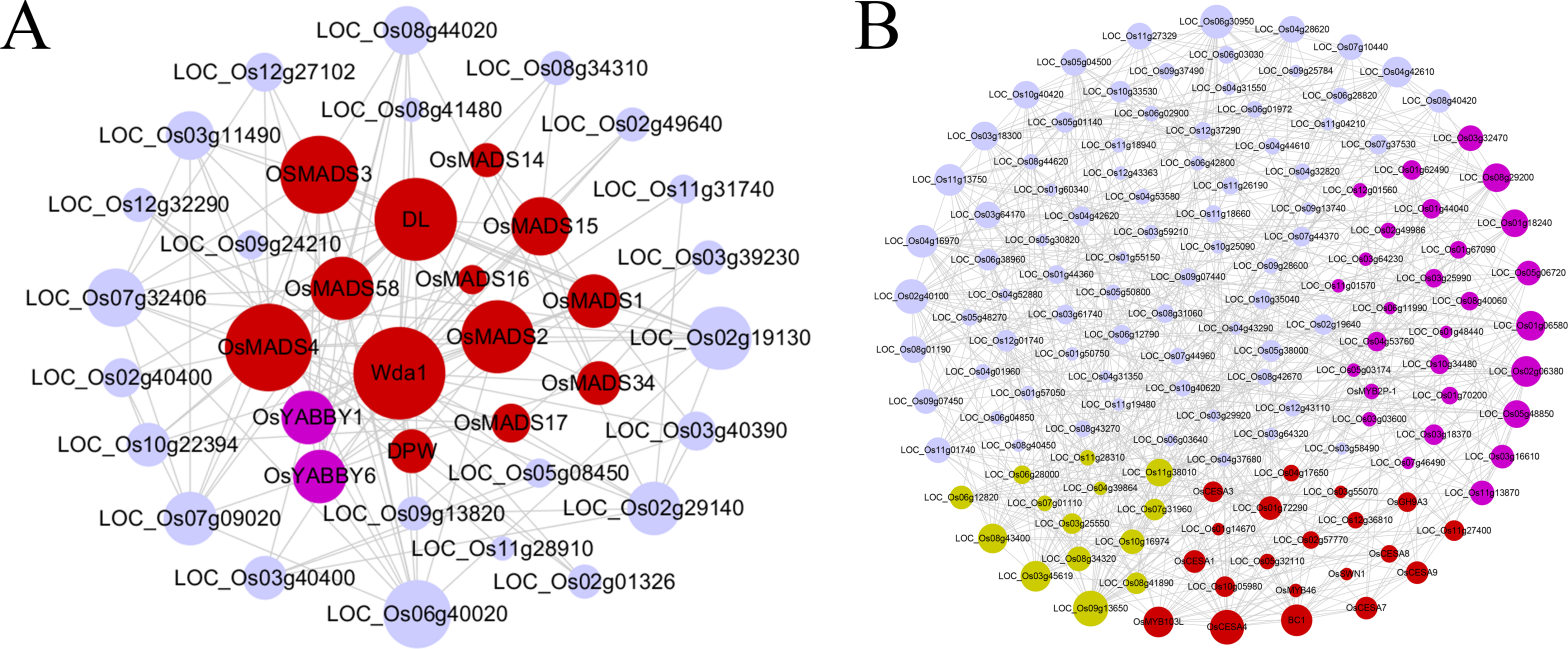
Subnetworks derived from the gene-guide approach. The subnetworks included all other nodes within two-layer connections from guide genes. A) *OsMADS16* involved in flower development; B) *OsCESA4* involved in cell wall biosynthesis. Within each subnetwork, red nodes represented the experimentally verified genes related to corresponding biological functions. Pink nodes indicated the genes whose *Arabidopsis* homologs were experimentally verified relating to the corresponding biological processes. Yellow nodes represented the potential function-related genes, and gray nodes denoted that the genes with unknown functions or annotated with irrelevant functions. The size of node was proportional to the number of connected genes.

In addition to the global co-expression neighboring gene functional analysis above, we used two examples below to demonstrate the stricter and more intuitive method of RNA-seq-based gene co-expression network analysis for inferring the gene functions. In flowering plants, floral organ development is a very important biological process. We therefore first selected a priori guide gene, *OsMADS16*, involved in the flower development, to obtain a co-expression subnetwork consisting of 37 closely connected neighbors within two-layer links from the guide genes (see Fig 4A and S6 Dataset). We found that 15 genes were involved in flower development process, with ~ 203-folds enrichment relative to whole genome. For example, 11 members of MADS-box family, which have been verified involving in determination of floral organ identity and development, were effectively captured in this subnetwork. Moreover, this subnetwork included the well-known genes, such as *DL*, *Wda1* and *DPW*, which have been experimentally validated to control the floral organ identity, anther and pollen development [47–49]. Interestingly, we found that two YABBY domain containing genes, *OsYABBY1* and *OsYABBY6*, were not annotated in floral organ development in rice, but their *Arabidopsis* homologs, *YABBY2* and *YABBY1*, were associated with the inflorescence meristem growth and regulation of floral organ development [50]. The links between the unannotated genes (gray nodes) and the known genes within a subnetwork provided clues for their associations with flower development. For example, *LOC_Os07g09020* was involved in the embryo development, whose links with *OsMADS3*, *OsMADS4* and *DL* enabled further targeted experimental validations.

Second, we used another guide-gene *OsCESA4* involved in cell wall metabolism to build a subnetwork (see Fig 4B and S6 Dataset). The resulting subnetwork was made up of 139 genes with ~96-folds enrichment, including 4 homologs of *OsCESA4*: *OsCESA1*, *OsCESA3*, *OsCESA7* and *OsCESA9*, and 14 other genes associated with cell wall metabolism. In addition, this subnetwork also captured 28 genes (pink nodes) whose *Arabidopsis thaliana* homologs were involved in cell wall metabolism. For example, *LOC_Os01g06580*, encoding a fasciclin domain containing protein, is a homolog of *AT5G03170* which was involved in secondary cell wall biogenesis. Two genes, *LOC_Os01g62490* and *LOC_Os03g16610*, are the laccase precursor proteins, both of them is the homologs of *LAC17*, a gene participated in the cell wall biogenesis. *AT1G09540*, an *Arabidopsis* homolog of two rice MYB family transcription factors, *LOC_Os05g04820* and *LOC_Os01g18240*, was involved in cell wall macromolecule metabolism and xylem development. We also noted that 14 genes labeled with yellow nodes, participating in carbohydrate metabolism, associating with microtubule or resembling to known cell wall genes in function domain, were recovered in this subnetwork. These genes can be the potential candidates for subsequent functional experiment investigation. Especially, known cell cycle genes *LOC_Os04g28620* and *LOC_Os04g53760* were also captured in this gene subnetwork, confirming that cell wall metabolism and cell cycle are two closely associated processes.

### Construction of regulatory subnetworks for gene function analysis

**Fig 5:**
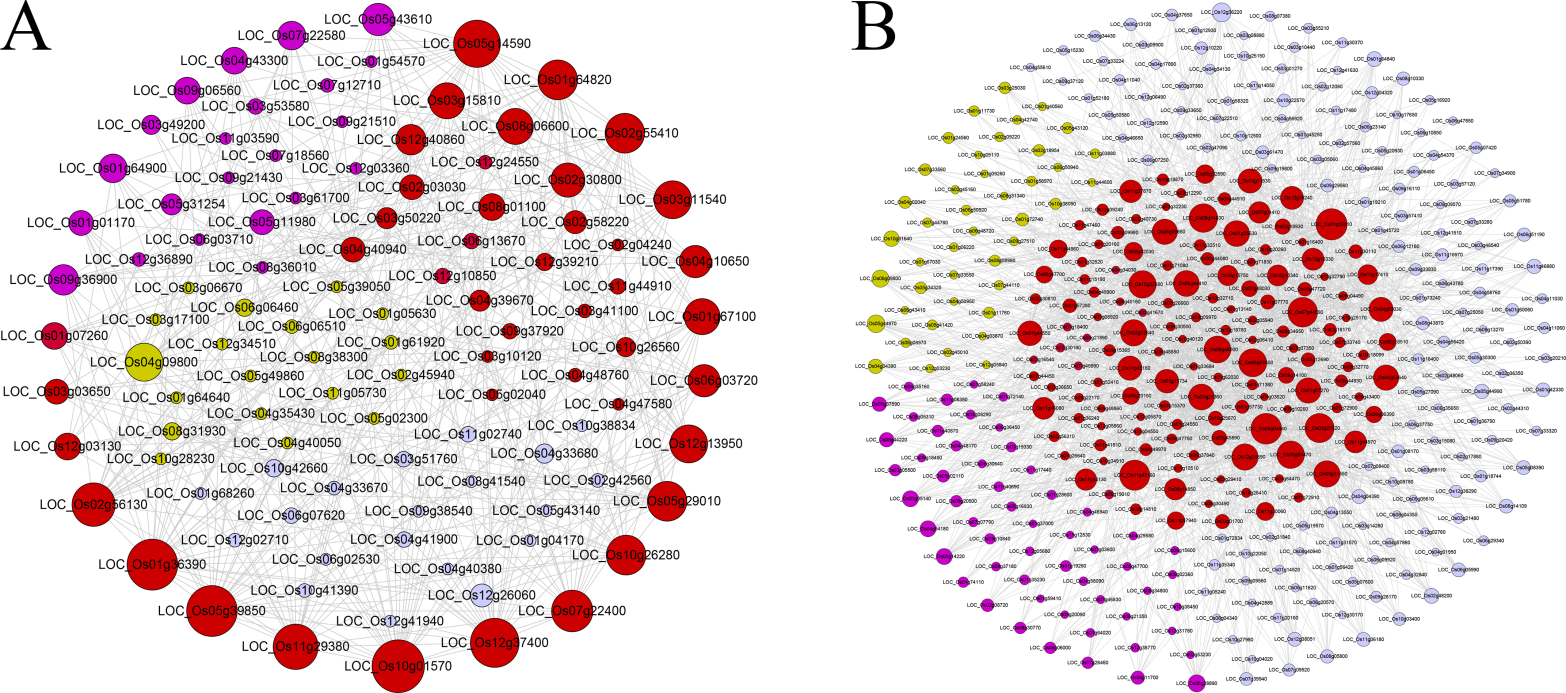
Subnetworks derived from the known *cis*-regulatory motif-guide approach. A) WTTSSCSS combined with the E2F transcription factors involved in cell cycle. B) TTGACY combined with the WRKY transcription factors involved in stress response. Within each subnetwork, red nodes represented the experimentally verified genes related to corresponding biological functions. Pink nodes indicated the genes whose *Arabidopsis thaliana* homologs were experimentally verified to be associated with the corresponding biological functions. Yellow nodes denoted the potential function-related genes. Gray nodes indicated that the genes with unknown functions or annotated with irrelevant functions. The size of node was proportional to the number of connected genes.

We explored the potential value of motif-guided analysis [5] in building regulatory network and finding functionally related genes using two examples. Cell cycle is a highly conserved biological process in higher eukaryotes. The process of G1 phase to S phase of the cell cycle is controlled by the E2F transcription factors, which binds to a conserved DNA motif WTTSSCSS (with “W” standing for “A” or “T” and “S” standing for “C” or “G”) [51]. We used this motif to retrieve 1093 genes from the rice network. Out of the 180 cell cycle genes annotated in rice (totally 55986 genes), 33 cell cycle genes were included in these 1093 genes, resulting in 9.4-folds enrichment. We used the cell cycle genes and the genes that were directly linked to them to form a regulatory network (totally 104 genes, Fig 5A and S6 Dataset). We have observed that numerous genes (red nodes in Fig 5A) encode proteins participating in regulation of cell cycle, DNA replication, chromatin dynamics and DNA repair. The currently known cell cycle genes included three cyclin genes, one E2F transcription factor, 9 DNA replication origin factors, two checkpoint regulators, 13 DNA replication and repair proteins and 10 other genes with unknown biochemical functions but were annotated playing important roles during cell cycle. In addition, this subnetwork also included 18 genes whose *Arabidopsis* homologs participated in regulation of cell cycle, DNA replication, DNA repair and chromatin dynamics. Also recovered were four genes including *LOC_Os01g64900*, *LOC_Os03g49200*, *LOC_Os07g18560* and *LOC_Os09g36900* whose *Arabidopsis* homologs did not have known biochemical functions but are involved in cell cycle. Although some genes were not annotated with direct participation of cell cycle, their molecular structures indicated their potential roles, for example, ATP-dependent RNA helicase (*LOC_Os11g44910*), ribonuclease H2 subunit B (*LOC_Os04g40050*), ribonuclease H2 subunit B (*LOC_Os04g40050*) and BRCA1 C Terminus domain containing protein (*LOC_Os08g31930*). All these genes can be candidate cell cycle genes for further investigation.

WRKY transcription factors play important roles in regulation of plant stress response by binding the W-box sequence TTGACY (with ‘‘Y” standing for ‘‘C” or ‘‘T”) [52,53]. Similarly, we extracted 1329 genes associating with W-box, from which a subset of 88 known stress response genes out of 996 genes relating to rice stress response process are found, achieving the enrichment of 3.72 folds. We constructed a regulatory network using the 88 genes and the genes with W-box that were directly linked to them (totally 389 genes, Fig 5B and S6 Dataset). This subnetwork included 172 genes that were regulated by different types of environmental stresses (red node). Among them, 138 rice genes and 34 homologs in *Arabidopsis* were annotated relating to abiotic and biotic stresses. The *Arabidopsis* homologs of many these genes have been experimentally verified to be involved in biological regulation of phosphate starvation, water deprivation and various stresses. In particular, 53 of 172 abiotic stress response genes whose *Arabidopsis* homologs reacted to the ethylene (ETH), abscisic acid (ABA), salicylic acid (SA) or jasmonic acid (JA), which was in accordance with the fact that WRKYs play roles in the plant abiotic stress [53]. Moreover, 36 genes have been confirmed played important roles in regulating plant immune responses to pathogens were also contained in this network (see S6 Dataset). This was completely supported by the transcriptional reprogramming network model of WRKY-mediated plant immune responses [54].

In addition, this subnetwork also included 8 genes whose *Arabidopsis* homologs were associated with the seed development, dormancy and germination. In agreement with the fact that the SA and ABA antagonizes gibberellin (GA)-promoted seed germination; six of these genes participated in the SA- and ABA-mediated signaling pathways [55]. Interestingly, three genes of *LOC_Os03g12290*, *LOC_Os01g24550* and *LOC_Os01g64470* involved in leaf senescence were also placed in this subnetwork, with *LOC_Os01g64470* involved in the SA- and JA-mediated signaling pathways, which was supported by the fact that the WRKYs participate in leaf senescence by modulating the JA and SA equilibrium [56]. This subnetwork successfully captured the W-box related genes that can facilitate further studies the functions of uncharacterized genes and help us to understand the mechanisms of plant responding to various stresses. Interestingly, we have found that miRNA-guide gene subnetwork can also effectively capture the functionally related genes (see S5 Text for details). Taken together, all these outcomes indicated that the rice RNA-seq-based gene co-expression network could be converted to highly reliable regulatory network for further studying gene regulations.

### Co-expression analysis of genes controlling the important agronomic traits

From the perspective of system biology, the phenotype of an organism is controlled by functionally linked genes involving in the related biological processes. Given the co-expressed genes tend to have related biochemical functions; we next want to use the co-expression relationships between genes to assign the agronomic traits for unknown genes. This is especially important for identifying the candidate genes in Quantitative Trait Loci (QTL) mapping, Genome-Wide Association Study (GWAS) or in reverse genetic studies. We collected 1031 known rice genes with the well-studied functions through wet lab experiments. For these genes, we found that 934 genes were expressed in our collected RNA-seq samples and 623 genes were contained in network with 12125 connections. To examine the potential capacity of our RNA-seq-based gene co-expression network for associating genes with agronomic traits, we compared the density of co-expression links within agronomic traits and between agronomic traits. We found that 262 co-expression links out of 88041 all possible links within common agronomic traits and 252 co-expression links out of 982302 all possible links between different agronomic traits were captured in our gene network, with ~11-fold enrichment of links within agronomic traits. In details, we observed that many agronomic traits whose genes were tightly linked together relative to the average link density of whole gene co-expression network (S2 Table). For example, an agronomic trait, source activity, measuring the capacity of making photosynthetic products; whose genes were highly linked in our network with an enrichment fold of 47.81 and a corrected *p*-value of 3.96E-117. In addition, genes associated with the culm leaf, panicle flower, eating quality and tolerance were also significantly connected. Moreover, we performed permutation test and discovered that the co-expression link densities between genes controlling the same agronomic traits were significantly larger than random control genes (see S2 Table for details). These results indicated that our gene network can be used to discover the gene related to important agronomic traits by co-expression links.

### Function discovering for lncRNA genes

**Fig 6:**
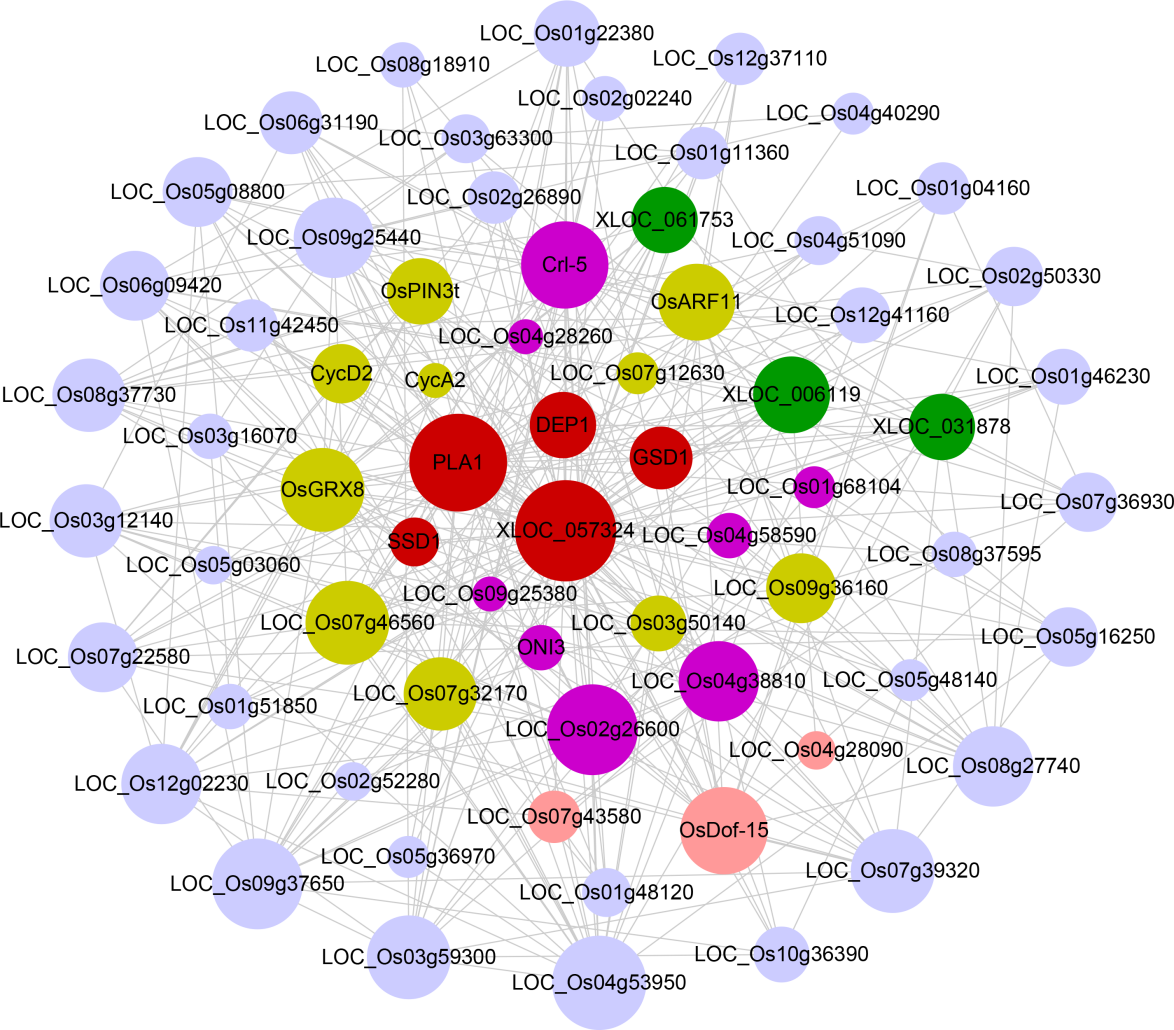
Co-expression subnetwork derived from guide-gene approach for *XLOC_057324* associated with panicle development and fertility. Within the subnetwork, red nodes represented the experimentally verified genes related to corresponding biological functions. Chrysoidine nodes represented transcription factors. Pink nodes indicated the genes whose *Arabidopsis thaliana* homologues were experimentally verified to be related to corresponding biological functions. Yellow nodes represented that the genes were potential function-related. Green nodes denoted the lncRNA genes and gray nodes indicated that the genes were function unknown or annotated with unrelated functions.

Long non-coding RNAs (lncRNAs) have been shown to play important roles in the kingdoms of plants and animals [57,58]. Given that our reconstructed RNA-seq-based co-expression network successfully associate genes with biological functions and phenotypes of interest, we next wished to discover the functions for uncharacterized lncRNA genes using network-based method. We downloaded the known lncRNAs of rice identified in previous studies [57]. We then combined these lncRNA genes with MSU 7.0 reference genes to establish co-expression network. The obtained network was composed of 24875 genes, containing 24014 protein-coding gene and 861 lncRNA genes connected by 1357039 edges. Compared with the previous network, 7692 novel protein-coding genes were captured and linked with 817 lncRNA genes. As there is no gold standard available to evaluate the predictive performance, we adopted gene-guide subnetwork analysis to illustrate the potential capacity of this network for lncRNA function discovering. We selected a well-studied lncRNA gene of *XLOC_057324*, which was verified to be involved in panicle development and fertility, to establish a gene subnetwork consisting of the two-layers co-expression neighborhoods (Fig 6 and S7 Dataset). Relative to whole genome annotations, this subnetwork achieved ~38 folds enrichment of functionally related genes. In this subnetwork, 4 genes including *SSD1*, *PLA1*, *DEP1* and *GSD1* related to the panicle development or fertility were captured. In addition, we found that seven genes whose *Arabidopsis* homologs participated in the meiosis, embryo development or reproductive process. According to the known annotations, some genes (yellow nodes) might be also involved in pollen development, such as, two cyclins *CYCA2* and *CYCD2*. Interestingly, 3 lncRNAs of *XLOC_061753*, *XLOC_006119* and *XLOC_031878* expressed in the reproductive organs were contained in this subnetwork. All these results were in good agreement with the experimentally verified role of *XLOC_057324*, indicating the powerful capacity of our approach in inferring the novel function of lncRNA genes.

### CircRNA gene identification and function analysis

CircRNA is an RNA molecule forming a covalently closed continuous loop that has been discovered in various species across the domains of life with many distinct sizes [59,60]. The potential functions of circRNAs are largely unknown and hard to investigate. Therefore, we tried to classify them through the gene co-expression network. We first identified 14325 circRNAs in rice derived from 5284 genes including 4609 protein-coding genes, 675 noncoding genes (see Materials and methods for details). 43 of these genes including 27 protein-coding genes and 16 non-coding genes produced the circRNAs with the percentage larger than 90% in at least one sample. We analyzed the distribution of the number of detected circRNAs and found that a majority of circRNAs were identified in one sample with relative small number of circRNAs were detected in more than 3 samples (Figure A in S13 Fig). Although a large number of circRNAs were detected in relative small number of RNA-seq samples, 63 circRNAs (transcribed from protein-coding genes), identified in more than 10 samples and were supported by more than 26 junctions reads, were captured in the co-expression network. Moreover, we found that the primary genes transcribing these circRNAs were not contained in the co-expression network. We predicted the functions of these circRNAs using GO enrichment analysis of their co-expression neighborhoods. Indeed, these circRNAs play a broad range of biological functions, for example, protein phosphorylation, ATP binding and photosynthesis (Figure B in S13 Fig). These results indicated that a great number of circRNAs play important biological roles but not are the transcriptional noise.

## Conclusion

The phenotypes of an organism are determined by the coordinated activity of many genes and gene products. To gain insight into the genetic foundation underlying the complex biological processes and phenotypes, we have developed a novel analytic pipeline for constructing genome-wide and high-quality RNA-seq-based co-expression network. We applied this pipeline to an important crop species rice. The co-expression links between genes were ranked by their confidence score, expression level and expression sample number. The thresholds of these measures can be selected as the indictors of co-expression reliability for targeted experimental validation. The detailed analysis of the topology properties of network demonstrated that the degree frequency distribution followed the truncated power-law and network structure was highly modular. Using the standard functional link data sets and bottom-up subnetwork analysis, we showed that the analysis pipeline can be effectively applied to study gene function and regulation. In particular, the potential application value of RNA-seq-based gene network for predicting biological roles of lncRNA and circRNA genes has been also well shown. Overall, our analysis provided new insights into the regulatory code underlying transcription control and a starting point for understanding the complex regulatory system.

Compared with the sequence-based gene function annotation, a great advantage of co-expression-based inference approach is that homologs are not required for a gene to receive a prediction. Naturally, it is the case when a novel function appears for a specific species and the genes participating in the new biological process do not have corresponding homologues in other species. This is especially interesting for the non-coding RNAs because only short regions of non-coding RNA transcripts are limited by sequence- or structure-specific interactions. Compared to the protein-coding gene, the difference in selection pressure makes it very difficult to find orthologous non-coding RNAs by their sequences. Our analysis of a rice lncRNA gene, *XLOC_057324*, suggested that the RNA-seq-based gene network can be effectively applied to annotate the functions of non-coding genome elements. Indeed, using BLAST search against NCBI Reference Sequence Database (RSD), we found that ~87% of rice unannotated rice genes did not have homologous genes in other species, respectively.

For RNA-seq-based gene co-expression network investigation, the creation of novel computational methods for building high-quality network poses a future fundamental challenge. Currently, only five methods, including Pearson’s Correlation Coefficient (PCCs) analysis, WGCNA, Canonical Correlation Analysis (CCA), SpliceNet and VCNet, have been used to build RNA-seq-based gene co-expression networks [16–18,61,62]. Three of these tools are indeed unable to be applied to the large number of RNA-seq samples owing to their inherent high computational complexity. For the uncertainty and complexity of mechanism models underlying RNA-seq data, we designed a novel ensemble-based inference pipeline to establish RNA-seq-based gene co-expression network. Our results showed that the committee of three inference methods provides more robust and less false positive and false negative results than each single algorithm. The improved performance of our ensemble inference method depends on the voting and rescoring scheme which can reduce the bias occurring in a single learning method and assign a higher confidence score to the interactions that are repeatedly retrieved by different methods. Indeed, the standpoint of aggregating the results of different algorithms has been adopted in various contexts and it has proven to be effective in a variety of applications [63–65].

In principle, co-expression meta-analysis can only detect co-regulations between the genes which are co-expressed constantly or are sometimes co-expressed but otherwise silent. However, many activation patterns of gene groups appear only under the specific experimental conditions but behave independently under the other conditions, which might not be captured by our method. To overcome this problem, high-efficiency bi-clustering methods can be integrated into our model to reveal the transcriptional gene interactions presented only under a specific subset of the experimental conditions [66]. Overall, our approach can further be improved by I) expanding our ensemble pipeline with other high-efficiency network inference methods [67], II) employing the more reasonable voting and rescoring schemes to generate the consensus networks.

## Supporting information

S1 Text Comprehensive analysis of rice RNA-seq transcriptome. (DOC)

S2 Text Network reliability and comprehensiveness evaluation by novel rice RNA-seq samples. (DOC)

S3 Text Network comparison. (DOC)

S4 Text Co-expression analysis of functionally related genes. (DOC)

S5 Text MiRNA-guided subnetwork analysis. (DOC)

S1 Fig Gene coverage by reads and correlation between the number of mapped reads and the number of expressed genes. (DOC)

S2 Fig Expression correlations between genes and between samples. (DOC)

S3 Fig Expression patterns and tissue-specific up-regulated genes. (DOC)

S4 Fig The density distributions of relative expression levels (*Z*-scores) of different types of tissues. (DOC)

S5 Fig The expression relationships between tissues and GO categories. (DOC)

S6 Fig The relative expression levels of genes along the chromosomes. (DOC)

S7 Fig The fraction distribution of different gene sets whose -log(*p*-value) exceeded the given significance level. (DOC)

S8 Fig Sliding window analysis of the co-expression of physically adjacent genes. (DOC)

S9 Fig Network topological analysis. (DOC)

S10 Fig The enrichment fold curve of co-expression links based on expression abundance ranking and expression sample number ranking. (DOC)

S11 Fig Assessment of the overlap and coherence between reconstructed rice RNA-seq-based gene co-expression networks and reference rice networks. (DOC)

S12 Fig Subnetworks derived from the miRNA-guide approach. (DOC)

S13 Fig The statistic results of circRNAs. (DOC)

S1 Table The representative functional categories in which the tissue-specific up-expressed genes were enriched. (DOC)

S2 Table The statistic table of agronomic traits whose genes were highly linked together in our network. (DOC)

S1 Dataset Overview table of primarily downloaded RNA-seq experiment samples. (XLS)

S2 Dataset Statistical table of RNA-seq samples included in our study. (XLS)

S3 Dataset Enrichment analysis table for tissue-specific up-expressed and down-expressed genes. (XLS)

S4 Dataset Statistical table of gene sets which are significantly co-expressed than random control sets. (XLS)

S5 Dataset Statistic table of co-expression link density significance analysis in the detailed functional categories. (XLS)

S6 Dataset The detailed functions table of genes contained in co-expression subnetworks. (XLS)

S7 Dataset The detailed functions table of genes contained in *XLOC_057324*-guided co-expression subnetwork. (XLS)
