# Supplementary material for: NetMiner-an ensemble pipeline for building genome-wide and high-quality gene co-expression network using massive-scale RNA-seq samples
Source: PLoS One. 2018 Feb 9;13(2):e0192613. doi: 10.1371/journal.pone.0192613 (PMC5806890; doi:10.1371/journal.pone.0192613)
Supplement: S1 Text — (DOC) [file pone.0192613.s001.doc]

**Comprehensive analysis of** **rice RNA-seq transcriptome**

After getting 6 expression matrices, correlation analysis of expression was performed between samples and between genes, and compared with which derived from publicly available microarray data sets. As showed in Figure A in S2 Fig, Pearson Correlation Coefficients (PCCs) between microarray samples were much higher when compared with RNA-seq samples, and VST data yielded highest inter-sample correlation among all RNA-seq data sets and was next to microarray levels. This was completely supported by a previous study in *Arabidopsis* . In contrast with the previous report, our results demonstrated that FPKM normalization reduced correlations between samples when compared with RAW data set . For the UQ, TMM and RLE normalization data sets, the inter-sample correlations were not change when compared with RAW data set since these methods have effect only on the correlations between genes (Figure A in S2 Fig). For the PCCs between genes, RAW, UQ and TMM data sets obtained a mono-modal distribution and almost perfectly overlapped with each other (Figure B in S2 Fig). As was in agreement with previous analysis , PCCs of VST data set represented a symmetrical bell-shaped distribution, which was similar to the distribution of PCCs derived from microarray data set (Figure B in S2 Fig). When compared with RAW data set, the PCC values were reduced by RLE and VST normalization, while they were increased by FPKM normalization (Figure B in S2 Fig). All data sets generated the correlations between gene expression profiles that were higher than random control distribution (Figure B in S2 Fig).

To gain insight into the transcriptional reprogramming controlling the development of different organs of rice, we further analyzed the gene expression patterns of different tissues. Our RNA-seq data sets showed that the expression patterns of tissue-specific genes were consistent with previously described expression patterns in literatures. For example, a well-studied gene *sps* encoding a sucrose-phosphate synthase have been showed exceptionally expressed in leaf, seedling and pollen , which was shown in our RNA-seq samples (Figure A in S3 Fig). We observed that the distributions of relative expression abundance for most of tissues did not deviate from whole organism obviously (S4 Fig). Although small expression variation was to be observed, as showed by the Principal Component Analysis (PCA), different tissues had disparate biomarkers (Figure B in S3 Fig). The morphological similarity of different tissues was well projected in first three Principle Components (PCs) space (explained 36% of the total variation) and showed in two developmental orientations. We found the developmental stage of tissues had a large effect on PCA distance between samples (Figure B in S3 Fig). While the root and callus samples, such as, were separated in the first three PCs space approximately, some root tip samples were closer to callus samples. Panicle was another example of deviating from the general trend which the organ identity dominated developmental stage. The booting stage panicles were prior adjacent to callus samples, whereas other panicle samples were preferential close to egg cells.

With the purpose of investigation of biological properties of tissue-specific expressed genes, we carried out the pair-wise comparison for each tissue using other samples as control to discover the up- or down-expressed genes (DESeq packages, *p*-value < 0.01 and fold-change > 5). Consistent with the large fluctuation of relative expression levels in pollen, more up-expressed genes were to be found in this tissue (Figure C in S3 Fig and S4 Fig). We performed function enrichment analysis separately for each set of up-regulated or down-regulated genes associated with the specific tissue against whole genome background using Fisher’s exact test. The enrichment results revealed the important biological associations (see S3 Dataset and S1 Table). For callus and panicle, up-regulated genes involved in IAA conjugate biosynthesis and flower development were enriched, respectively. With regard to pollen, up-regulated genes were associated with DNA replication, homologous recombination and signal transduction. In terms of root, up-regulated genes participated in the manganese ion binding, copper ion binding and defense response. As for shoot, up-expressed genes were involved in photosynthesis, chloroplast and photorespiration. Unexpectedly, leaf-specific up-expressed genes were not enriched in photosynthesis. This might be closely related to that the leaf-specific up-regulated genes which were overlapped by shoot-specific up-regulated genes, since leaf is only a partition of shoot. Analysis of down-regulated genes demonstrated that the genes involved in photosynthesis were highly enriched in root samples (S3 Dataset). Overall, these results demonstrated that different tissues had the distinct biomarkers that were consistent with their specific biological functions.

We targeted 9 Gene Ontology (GO) categories and evaluated their relative expression activity in different types of tissues. It was biologically plausible that genes relating to reproduction, replication, pollination, and signaling were expressed [preferentially](javascript:void(0);) in pollen and that genes related to flower development were expressed highly in panicles (Figure A in S5 Fig). Similarly, it was also not surprising that genes related to photosynthesis were up-expressed in shoot, seedling and leaf (Figure B in S5 Fig). In addition, it was also reasonable that genes related to replication, transcription and translation were highly expressed in callus and panicle, whereas genes associated with cell wall metabolism were highly expressed in shoot, stem and root samples (Figure A in S5 Fig). In addition to analyzing the relative expression activities of different GOs in specific tissue types, we also scanned the whole genome for exploring the overall change of transcriptional activity. As expected, we found that the genes located in centromere regions had obviously reduced expression (S6 Fig).

Previous co-expression meta-analyses using microarrays demonstrated that the genes participating in the same pathways or being part of the same protein complexes are often co-regulated, and that clusters of genes with similar biological functions often exhibit the expression patterns which are correlated under a large number of experimental conditions . To examine whether this guilt-by-association relationship can be uncovered by our rice RNA-seq samples, we analyzed the expression pattern similarity for the genes assigned to the predefined gene set categories, including GOs, KEGG pathways, OryzaCyc pathways, microRNA target gene families, transcription factor families, paralogous gene families and Tos17 mutation phenotypes (S4 Dataset). The significance of co-expression within a given category was quantified by computing the distribution of pair-wise expression correlation within a given gene set category, and by comparing it to the distribution of random control gene set of the same size (see below for details). As showed in S7 Fig, many categories received a remarkable *p*-value and were significantly co-expressed than random sets, indicating that, in the rice RNA-seq data, functionally related genes were often to be co-expressed. It has also been reported that the genes of adjacent physical location tend to be co-expressed . Here, we examined the co-expression between the genes located in adjacent chromosome regions based on the sliding window analysis (see below for details). To obtain an appropriate window size, we repeated the analysis using the windows of various sizes. As showed in S8 Fig, the window size increased from 5 to 50 genes, the number of net genes (defined as the number of genes within the co-expressed adjacent groups) augmented rapidly. When the window size increased from 50 to 100 genes, the number of net genes became stable. This indicated that most groups included about 50 genes; we therefore used 50 genes as an optimal size for subsequent analysis. We found that many physical adjacency gene groups were more likely to be co-expressed than would be expected by chance, even though after the paralogous genes physically adjacent were removed (S8 Fig). These results indicated that many genes in the close physical proximity or sharing similar functions have been co-selected for performing specific biological activities that were necessary for the survival of the organism. To sum up, all outcomes above revealed that these 348 RNA-seq samples had high-quality and the co-expression links between genes which can be applied to infer their functional relationships.

**Gene co-expression analysis of** **predefined functional categories and adjacent chromosome regions**

The extent of co-expression of genes assigned to the same functional categories was quantified by a *p*-value of *t*-test. In details, the pair-wise PCCs were evaluated for all gene pairs within each category and random control sets, using all the experimental conditions in our RNA-seq data set. The resulting distributions were compared with a background distribution of 10,000 randomly chosen gene pairs using the *t*-test. The *p*-values were calculated for all predefined categories, as well as for random control sets of the same size distribution. For the physically adjacent genes, we calculated the pair-wise PCCs of expression patterns for all possible gene pairs that were within *n* genes (an *n*-gene window) and within random control set using the sliding window analysis. Significance (*p*-value) of the gene co-expression was estimated using the same method as predefined functional categories. To test whether paralogous genes account for the co-expression of physically adjacent genes, we searched for cases in which homologs were near to each other in each sliding window. If a gene showed homology to another gene within a sliding window, it was removed from the data set. The “homologues removed” gene data set was subjected to the algorithm above.

**References**

1. Giorgi FM, Del Fabbro C, Licausi F (2013) Comparative study of RNA-seq-and Microarray-derived coexpression networks in Arabidopsis thaliana. Bioinformatics 29: 717-724.

2. Chávez Bárcenas AT, Valdez Alarcón JJ, Martı́nez Trujillo M, Chen L, Xoconostle Cázares B, et al. (2000) Tissue-specific and developmental pattern of expression of the rice sps1 gene. Plant Physiol 124: 641-654.

3. Eisen MB, Spellman PT, Brown PO, Botstein D (1998) Cluster analysis and display of genome-wide expression patterns. Proc Natl Acad Sci USA 95: 14863-14868.

4. Kim SK, Lund J, Kiraly M, Duke K, Jiang M, et al. (2001) A gene expression map for Caenorhabditis elegans. Science 293: 2087-2092.

5. Ihmels J, Bergmann S, Barkai N (2004) Defining transcription modules using large-scale gene expression data. Bioinformatics 20: 1993-2003.

6. Schmid M, Davison TS, Henz SR, Pape UJ, Demar M, et al. (2005) A gene expression map of Arabidopsis thaliana development. Nat Genet 37: 501-506.

7. Caron H, van Schaik B, van der Mee M, Baas F, Riggins G, et al. (2001) The human transcriptome map: clustering of highly expressed genes in chromosomal domains. Science 291: 1289-1292.

8. Cohen BA, Mitra RD, Hughes JD, Church GM (2000) A computational analysis of whole-genome expression data reveals chromosomal domains of gene expression. Nat Genet 26: 183-186.
