# Supplementary material for: NetMiner-an ensemble pipeline for building genome-wide and high-quality gene co-expression network using massive-scale RNA-seq samples
Source: PLoS One. 2018 Feb 9;13(2):e0192613. doi: 10.1371/journal.pone.0192613 (PMC5806890; doi:10.1371/journal.pone.0192613)
Supplement: S2 Text — (DOC) [file pone.0192613.s002.doc]

**Network reliability and comprehensiveness evaluation by novel rice RNA-seq samples**

To evaluate whether a large fraction of potential gene interactions was represented by 348 collected rice RNA-seq samples, 618 novel RNA-seq samples downloaded from NCBI Sequence Read Archive (accessed on February 15, 2016) were used to construct another gene co-expression network using our ensemble method. We found that the 32% of the co-expression links in primary rice network also existed in this network. This was comparable to the outcome of previous study, which showed 41% common interactions between cross-species conserved gene co-expression networks derived from different microarray data sets [1]. Additionally, we also analyzed the consistency of module assignment by considering whether two genes were concurrently assigned to the same modules in the primary network and the updated network. We found 75% gene pairs with the same module assignment. These results indicated that 1) the majority of general transcriptional interactions were already established reliably with 348 rice RNA-seq samples; 2) integrating the new RNA-seq samples derived from certain experimental conditions were required to discover the novel transcriptional interactions.

**Reference**

1. Stuart JM, Segal E, Koller D, Kim SK (2003) A gene-coexpression network for global discovery of conserved genetic modules. Science 302: 249-255.
