# Supplementary material for: NetMiner-an ensemble pipeline for building genome-wide and high-quality gene co-expression network using massive-scale RNA-seq samples
Source: PLoS One. 2018 Feb 9;13(2):e0192613. doi: 10.1371/journal.pone.0192613 (PMC5806890; doi:10.1371/journal.pone.0192613)
Supplement: S3 Text — (DOC) [file pone.0192613.s003.doc]

**Network comparison**

We evaluated in detail the overlap of our co-expression network with protein-protein interaction network and probabilistic functional gene network, and the coherence of co-expression links with GO and pathway networks, which were obtained by linking all possible gene pairs within each functional category. Note that we only selected the edges connecting the shared genes between our co-expression network and each reference network for performance evaluation. For each different ratio of co-expression links obtained by different cutoff scores, we calculated several parameters of performance, including the Accuracy, Matthews Correlation Coefficient (MCC), Receiver Operating Characteristic (ROC) curve and Precision Recall (PR) curve. For all these reference gene networks, the predictive accuracy of co-expression inference was increased with the improving of cutoff score (Figure A in S11 Fig). As demonstrated by Matthews Correlation Coefficient (MCC); our gene network was always a positive predictor (MCC>0) for all reference networks, while the overlaps and coherences between our network and the reference networks were relatively low (Figure B in S11 Fig). All these results revealed that our network was indeed realistic, with the accuracy increasing with the network stringency, verifying the hypothesis that RNA-seq-based inference of gene co-expression was better than a random predictor of authentic biological relationships. We also observed that the RNA-seq-based gene network achieved higher performance for probabilistic functional gene network and pathway network, when compared with protein-protein interaction network and GO network (S11 Fig).
