# Supplementary material for: NetMiner-an ensemble pipeline for building genome-wide and high-quality gene co-expression network using massive-scale RNA-seq samples
Source: PLoS One. 2018 Feb 9;13(2):e0192613. doi: 10.1371/journal.pone.0192613 (PMC5806890; doi:10.1371/journal.pone.0192613)
Supplement: S4 Text — (DOC) [file pone.0192613.s004.doc]

**Co-expression analysis of functionally related genes**

We examined whether the genes within the same functional categories were tightly connected together in our networks. We carried out a statistical analysis to compare the co-expression links density of within the same functional categories to the density of links of the whole network. For GO terms within Biological Process (BP), Molecular Function (MF) and Cellular Component (CC), we found that, overall, the densities of co-expression links within the same biological categories were substantially higher than the average densities of links in whole networks (*p*-value=0, Fisher’s exact test). In details, there are 14.63% categories whose genes were significantly more linked than the whole network (see S5 Dataset for details). These results suggested that I) in a certain extent, functionally associated genes were linked significantly to form the transcriptional units; II) the genes within some GO categories did not completely correspond to a unit and several co-expression subgroups might be exist. Interestingly, we also found that the genes located in adjacent physical region were significantly linked in our network than the random control one with the *p*-value of 1.74E-5 (paired *t*-test).
