# Supplementary material for: NetMiner-an ensemble pipeline for building genome-wide and high-quality gene co-expression network using massive-scale RNA-seq samples
Source: PLoS One. 2018 Feb 9;13(2):e0192613. doi: 10.1371/journal.pone.0192613 (PMC5806890; doi:10.1371/journal.pone.0192613)
Supplement: S5 Text — (DOC) [file pone.0192613.s005.doc]

**MiRNA-guided subnetwork analysis**

We used two miRNAs, osa-miR156 and osa-miR396, to capture the functionally related genes using miRNA target gene enrichment analysis, which was similar with motif enrichment analysis [1]. We observed that many genes involving in the cell division and organ development were captured in this subnetwork, for example, two TCP transcription factors of *LOC_Os01g55100* and *LOC_Os11g07460* (see S12 Fig and S6 Dataset). Meanwhile, we also found that many genes associating with rice stress tolerance were placed in the subnetwork of *osa-miR156*, such as, a WRKY transcription factor, *LOC_Os10g18099* (see S12 Fig and S6 Dataset). These obtained results well confirmed the biological roles of these two miRNAs [2-4].

**References**

1. Ma S, Shah S, Bohnert HJ, Snyder M, Dinesh-Kumar SP (2013) Incorporating motif analysis into gene co-expression networks reveals novel modular expression pattern and new signaling pathways. PLoS Genet 9: e1003840.

2. Wu G, Park MY, Conway SR, Wang J-W, Weigel D, et al. (2009) The sequential action of miR156 and miR172 regulates developmental timing in Arabidopsis. Cell 138: 750-759.

3. Rodriguez RE, Mecchia MA, Debernardi JM, Schommer C, Weigel D, et al. (2010) Control of cell proliferation in Arabidopsis thaliana by microRNA miR396. Development 137: 103-112.

4. Stief A, Altmann S, Hoffmann K, Pant BD, Scheible W-R, et al. (2014) Arabidopsis miR156 regulates tolerance to recurring environmental stress through SPL transcription factors. Plant Cell 26: 1792-1807.
