# Supplementary material for: NetMiner-an ensemble pipeline for building genome-wide and high-quality gene co-expression network using massive-scale RNA-seq samples
Source: PLoS One. 2018 Feb 9;13(2):e0192613. doi: 10.1371/journal.pone.0192613 (PMC5806890; doi:10.1371/journal.pone.0192613)
Supplement: S1 Fig — (DOC) [file pone.0192613.s006.doc]

**
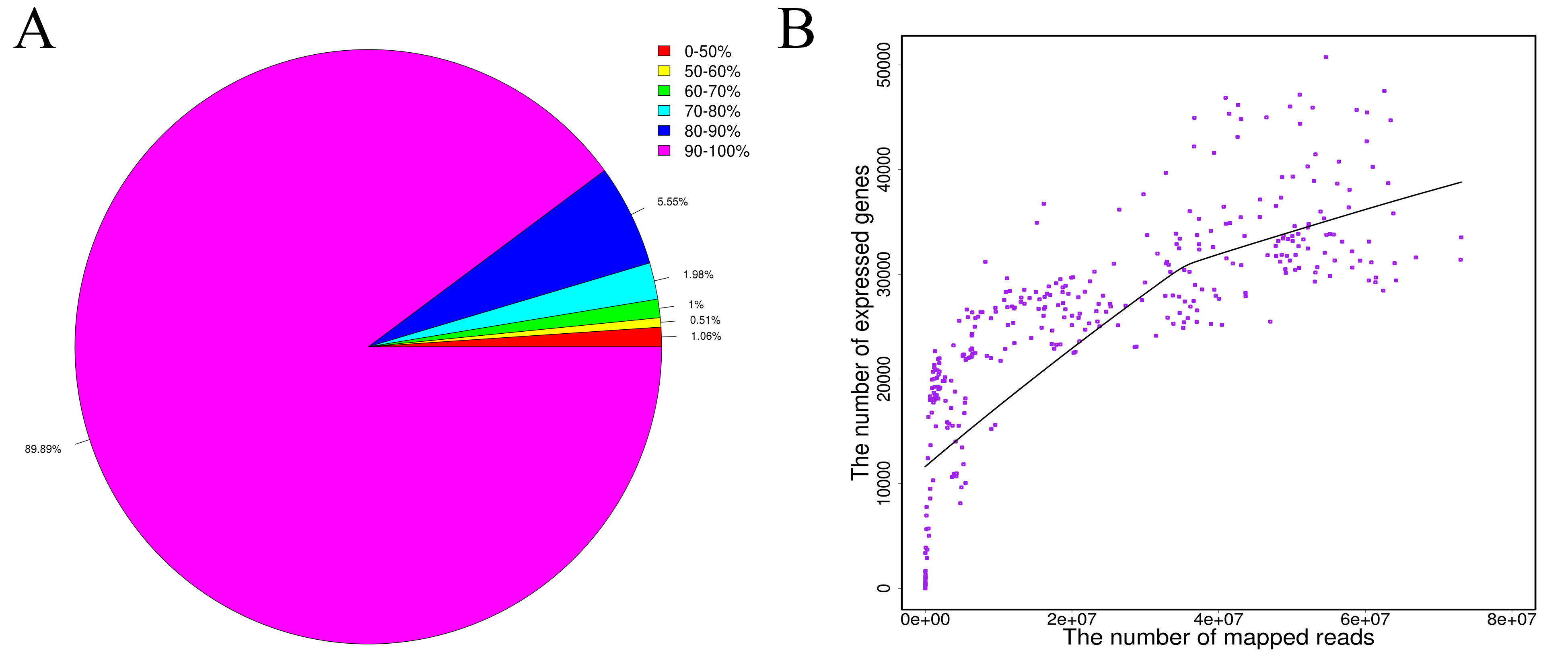
**

**S1 Fig** Gene coverage by reads and correlation between the number of mapped reads and the number of expressed genes. A) Gene coverage by reads, calculated as the percentage of the gene region covered by reads out of the total gene length. B) The association between the number of mapped reads and the number of expressed genes
