# Supplementary material for: NetMiner-an ensemble pipeline for building genome-wide and high-quality gene co-expression network using massive-scale RNA-seq samples
Source: PLoS One. 2018 Feb 9;13(2):e0192613. doi: 10.1371/journal.pone.0192613 (PMC5806890; doi:10.1371/journal.pone.0192613)
Supplement: S2 Fig — (DOC) [file pone.0192613.s007.doc]

**
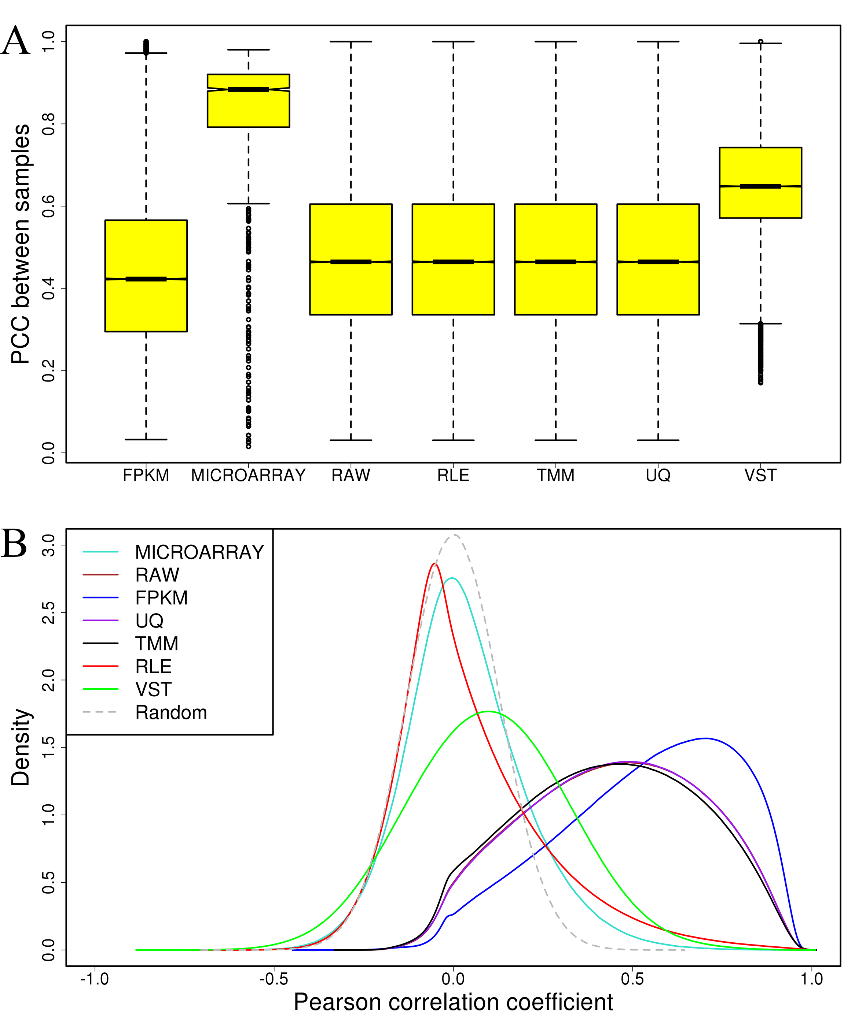
**

**S2 Fig** Expression correlations between genes and between samples. A) The box plots of PCCs between samples. B) The density distributions of PCCs between genes
