# Supplementary material for: NetMiner-an ensemble pipeline for building genome-wide and high-quality gene co-expression network using massive-scale RNA-seq samples
Source: PLoS One. 2018 Feb 9;13(2):e0192613. doi: 10.1371/journal.pone.0192613 (PMC5806890; doi:10.1371/journal.pone.0192613)
Supplement: S3 Fig — (DOC) [file pone.0192613.s008.doc]

**
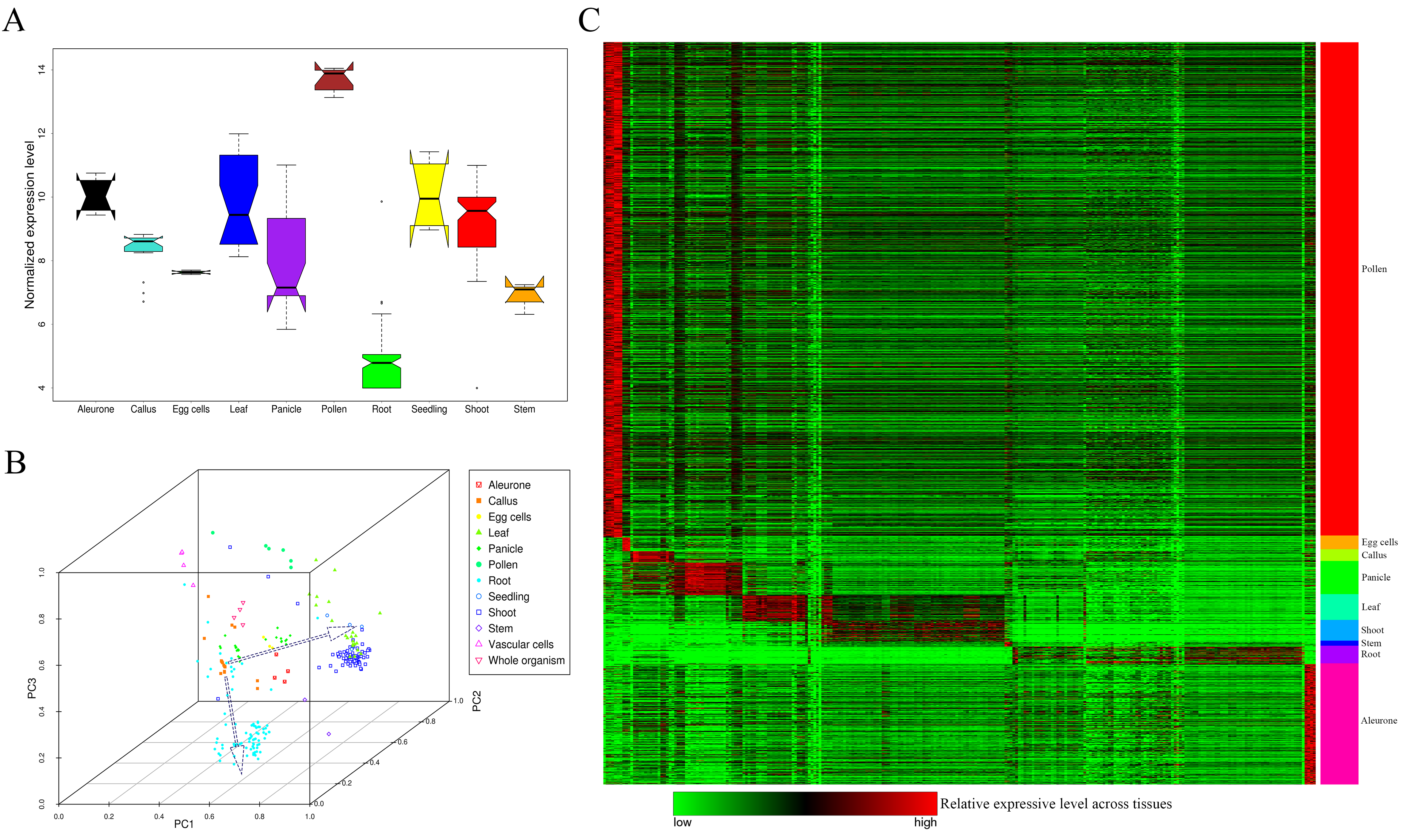
**

**S3 Fig** Expression pattern and tissue-specific up-regulated genes. A) The expression pattern of sucrose-phosphate synthase (*sps*) in different tissues. B) The distribution of different tissue samples in three-dimensional space made up of first three principle components of PCA. C) Heat map plot of the tissue-specific up-expressed marker genes obtained by DESeq. Since it has been demonstrated that VST data set is more appropriate for PCA analysis [1], our provided the results obtained using VST data set. Other data sets given the similar results

**Reference**

1. Anders S, Huber W (2010) Differential expression analysis for sequence count data. Genome Biol 11: R106.
