# Supplementary material for: NetMiner-an ensemble pipeline for building genome-wide and high-quality gene co-expression network using massive-scale RNA-seq samples
Source: PLoS One. 2018 Feb 9;13(2):e0192613. doi: 10.1371/journal.pone.0192613 (PMC5806890; doi:10.1371/journal.pone.0192613)
Supplement: S4 Fig — (DOC) [file pone.0192613.s009.doc]

**
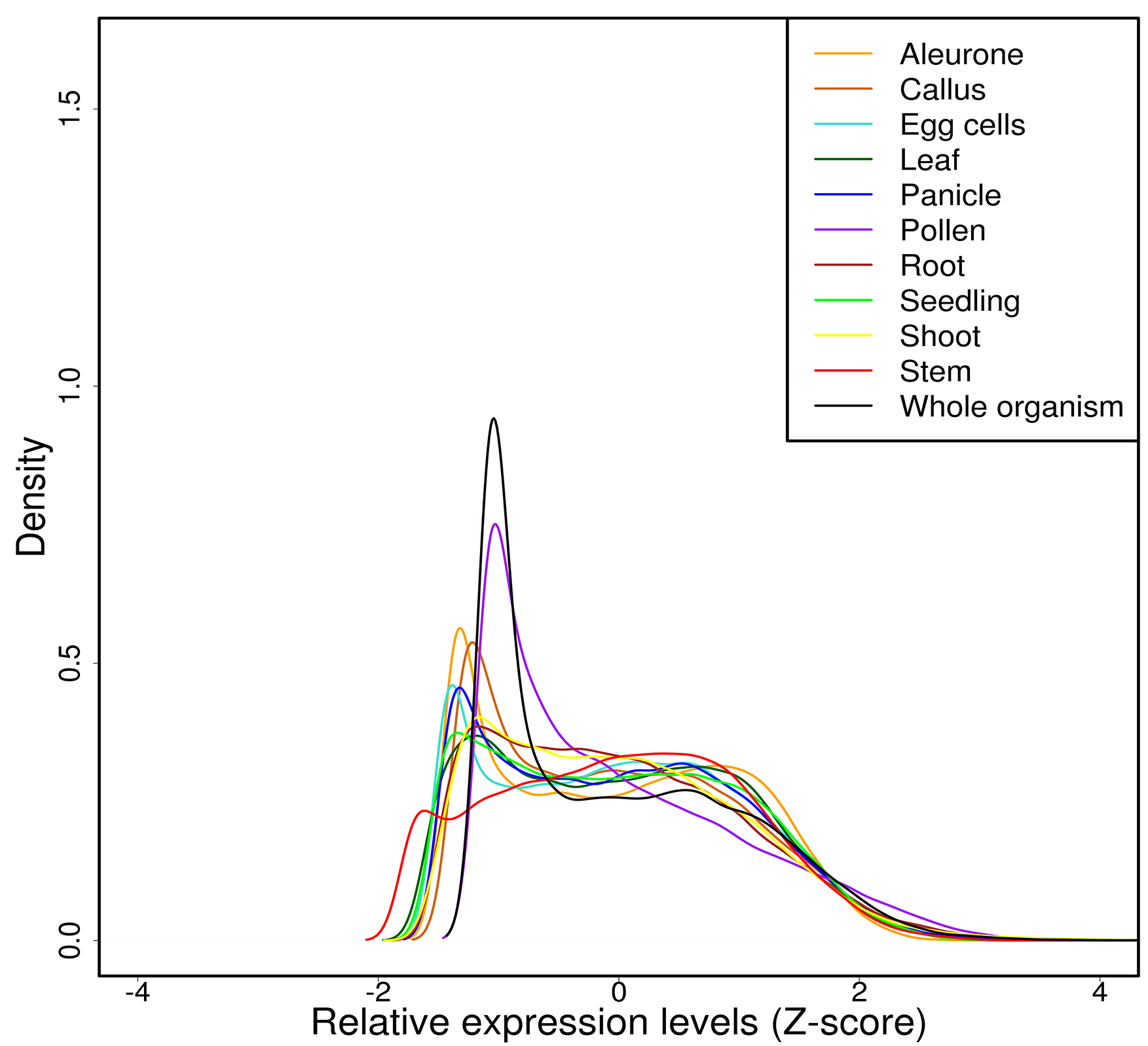
**

**S4 Fig** Density distributions of relative gene expression levels (*Z*-scores) of different tissues. The *Z*-scores were obtained using FPKM data set. Other data sets given the similar results
