# Supplementary material for: NetMiner-an ensemble pipeline for building genome-wide and high-quality gene co-expression network using massive-scale RNA-seq samples
Source: PLoS One. 2018 Feb 9;13(2):e0192613. doi: 10.1371/journal.pone.0192613 (PMC5806890; doi:10.1371/journal.pone.0192613)
Supplement: S5 Fig — (DOC) [file pone.0192613.s010.doc]

**
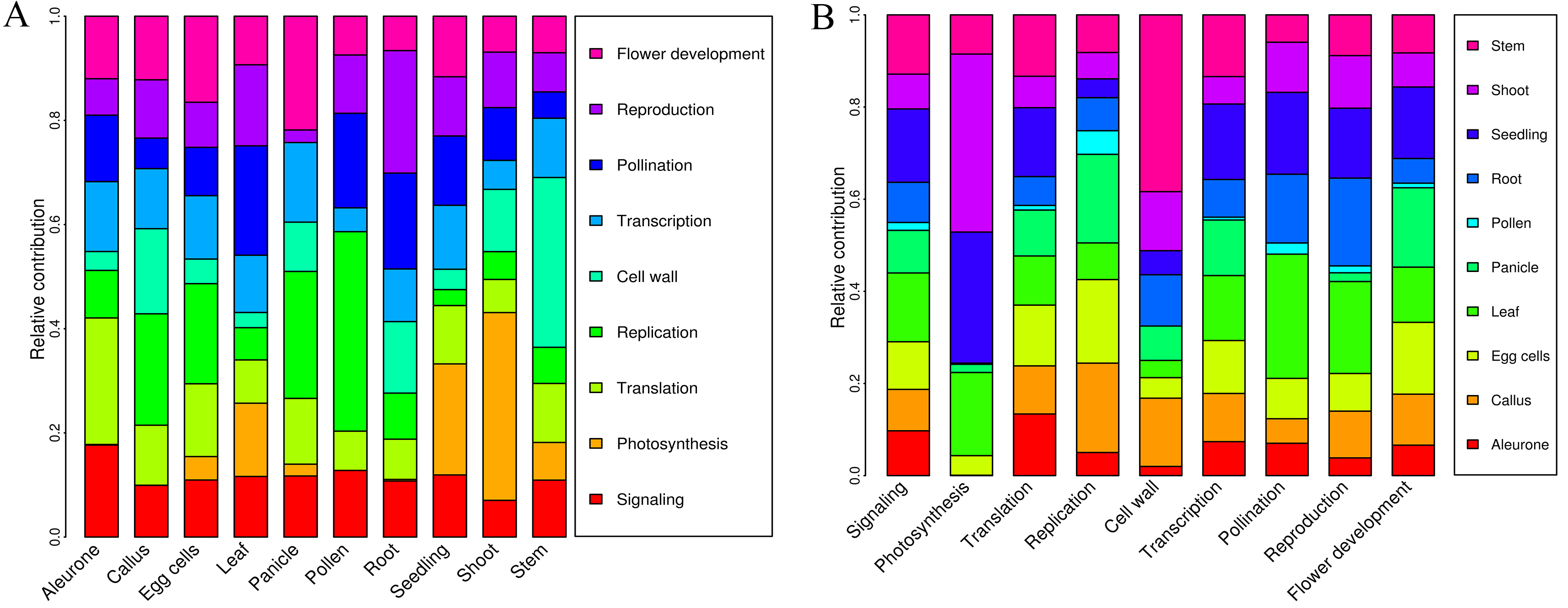
**

**S5 Fig** The expression relationships between tissues and GO categories. A) Relative activities of GO categories in different tissue types. B) Relative activities of the tissue types in different GO categories. The average expression levels of the genes within each GO category was used to obtain the relative activity. The relative activities were calculated using FPKM data set. Other data sets given the similar results
