# Supplementary material for: NetMiner-an ensemble pipeline for building genome-wide and high-quality gene co-expression network using massive-scale RNA-seq samples
Source: PLoS One. 2018 Feb 9;13(2):e0192613. doi: 10.1371/journal.pone.0192613 (PMC5806890; doi:10.1371/journal.pone.0192613)
Supplement: S6 Fig — (DOC) [file pone.0192613.s011.doc]

**
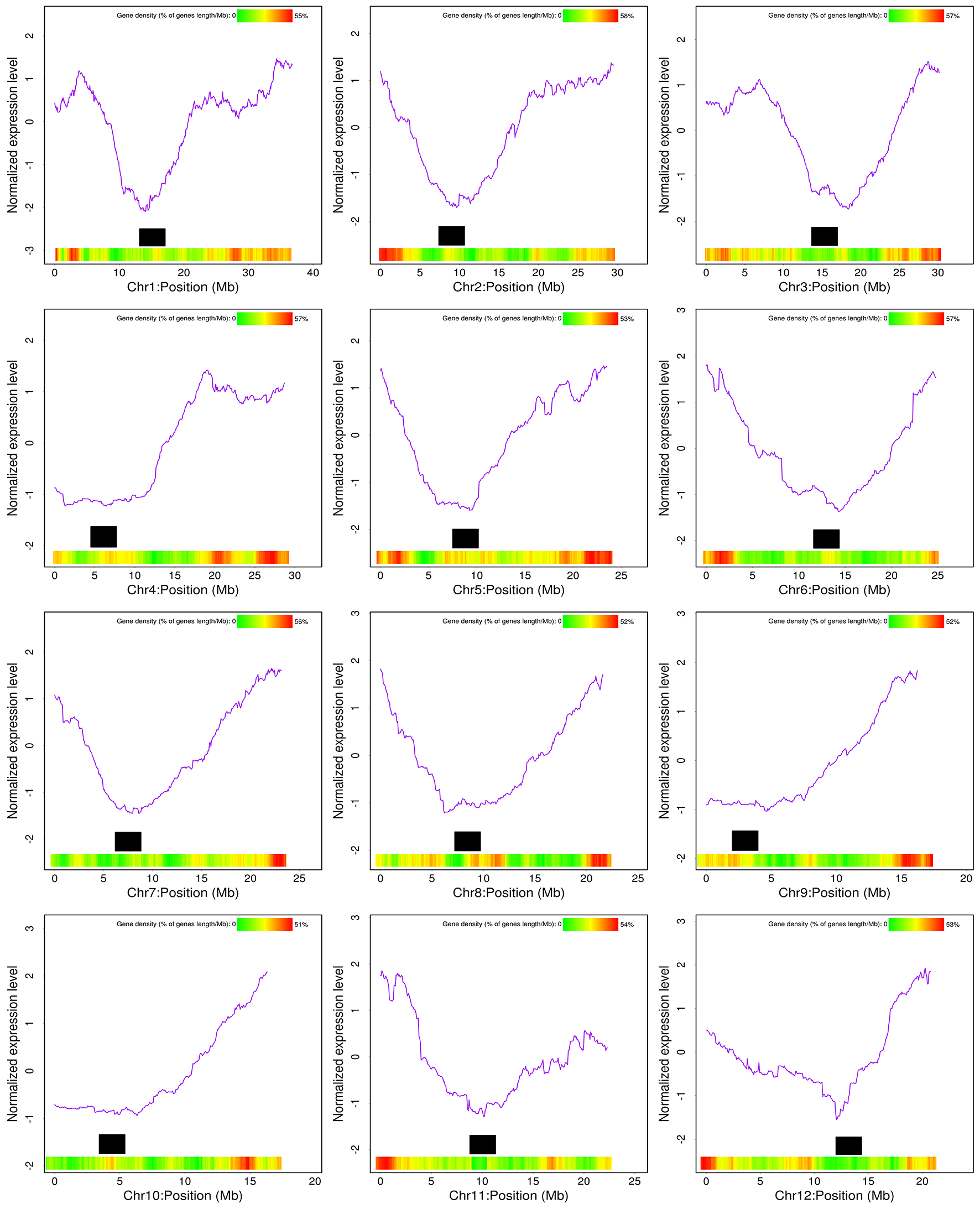
**

**S6 Fig** The relative expression levels of genes along the chromosomes; sliding window analysis of averaged gene expression levels along each chromosome with the window size of 1000 genes and step size of 10 genes. The relative expression levels were obtained using FPKM data set. Other data sets given the similar results
