# Supplementary material for: NetMiner-an ensemble pipeline for building genome-wide and high-quality gene co-expression network using massive-scale RNA-seq samples
Source: PLoS One. 2018 Feb 9;13(2):e0192613. doi: 10.1371/journal.pone.0192613 (PMC5806890; doi:10.1371/journal.pone.0192613)
Supplement: S7 Fig — (DOC) [file pone.0192613.s012.doc]

**
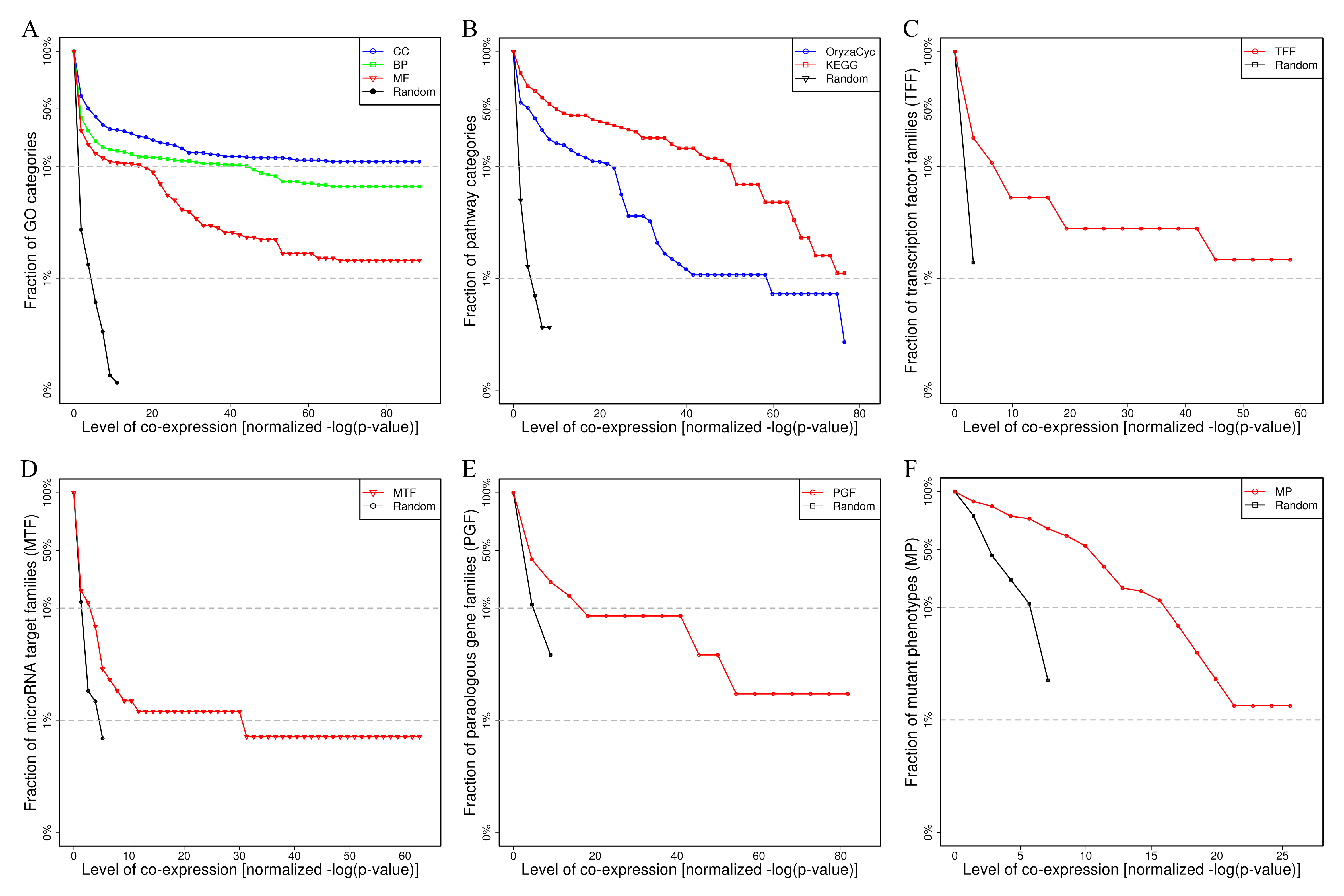
**

**S7 Fig** The fraction distribution of different gene sets whose -log(*p*-value) exceeded the given threshold value. The *p*-values were obtained by comparing the distribution of pair-wise PCCs between genes within the category to the distribution of random control set of the same size. A) GO categories vs random control categories, CC: Cellular Component, BP: Biological Process, MF: Molecular Function; B) pathway categories vs random control categories, OryzaCyc: pathways from OryzaCyc database, KEGG: pathways from PlantGSEA database; C) transcription factor families (TFF) vs random control categories; D) microRNA target families (MTF) vs random control categories; E) paralogous gene families (PGF) vs random control categories; F) Tos17 mutation phenotypes (MP) vs random control categories. Note our analysis were carried out using FPKM data set. The other data sets given the similar results
