# Supplementary material for: NetMiner-an ensemble pipeline for building genome-wide and high-quality gene co-expression network using massive-scale RNA-seq samples
Source: PLoS One. 2018 Feb 9;13(2):e0192613. doi: 10.1371/journal.pone.0192613 (PMC5806890; doi:10.1371/journal.pone.0192613)
Supplement: S8 Fig — (DOC) [file pone.0192613.s013.doc]

**
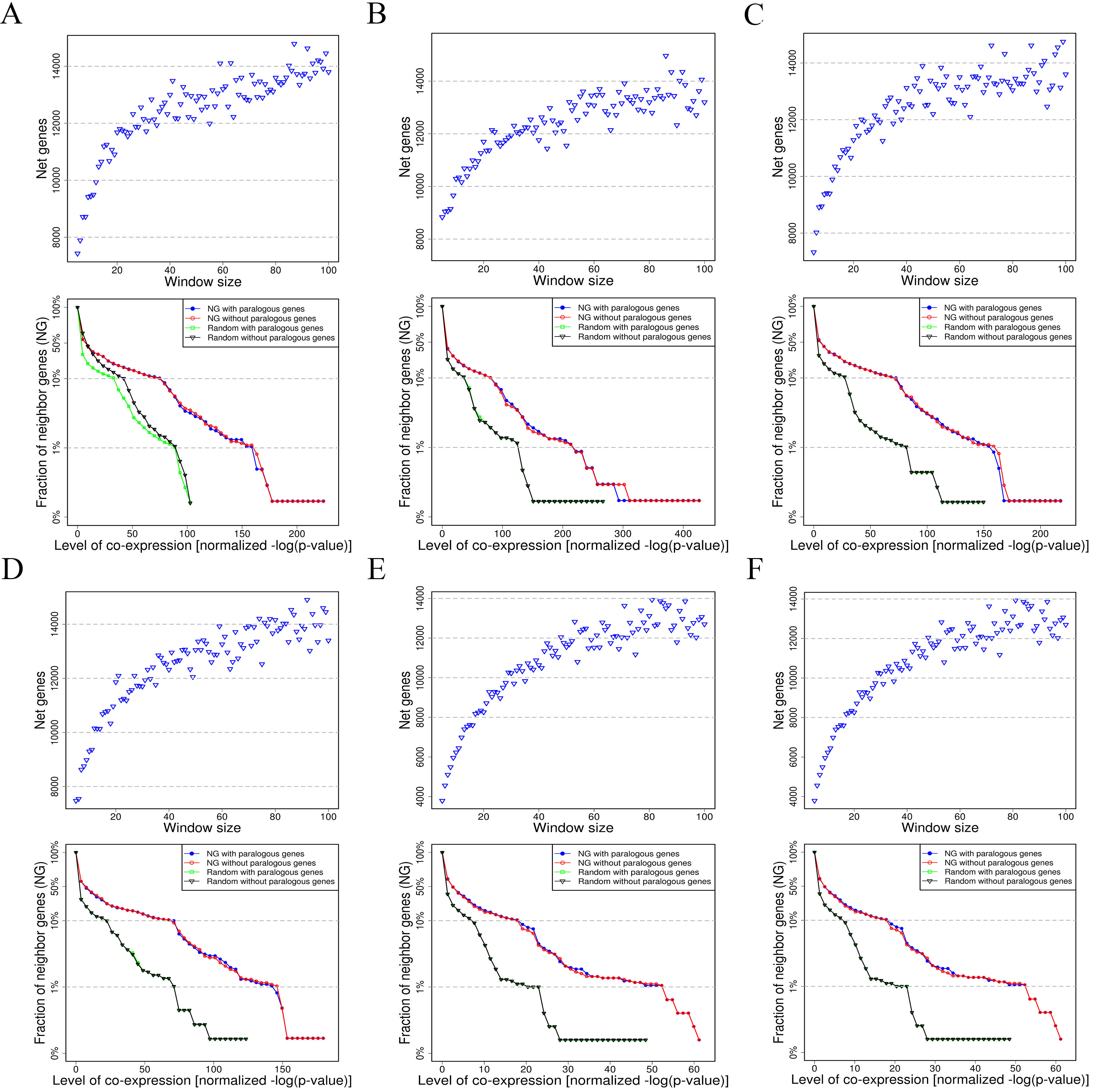
**

**S8 Fig** Sliding window analysis of the co-expression of physically adjacent genes. A, RAW data set. B, FPKM data set. C, UQ data set. D, TMM data set. E, RLE data set. F, VST data set. For each sub-figure (for example A), the top section described the distribution of the number of net genes when different window sizes were used, the bottom section described the fraction distribution of physically adjacent gene groups and random control groups whose -log(*p*-value) exceeded a threshold value
