# Supplementary material for: NetMiner-an ensemble pipeline for building genome-wide and high-quality gene co-expression network using massive-scale RNA-seq samples
Source: PLoS One. 2018 Feb 9;13(2):e0192613. doi: 10.1371/journal.pone.0192613 (PMC5806890; doi:10.1371/journal.pone.0192613)
Supplement: S9 Fig — (DOC) [file pone.0192613.s014.doc]

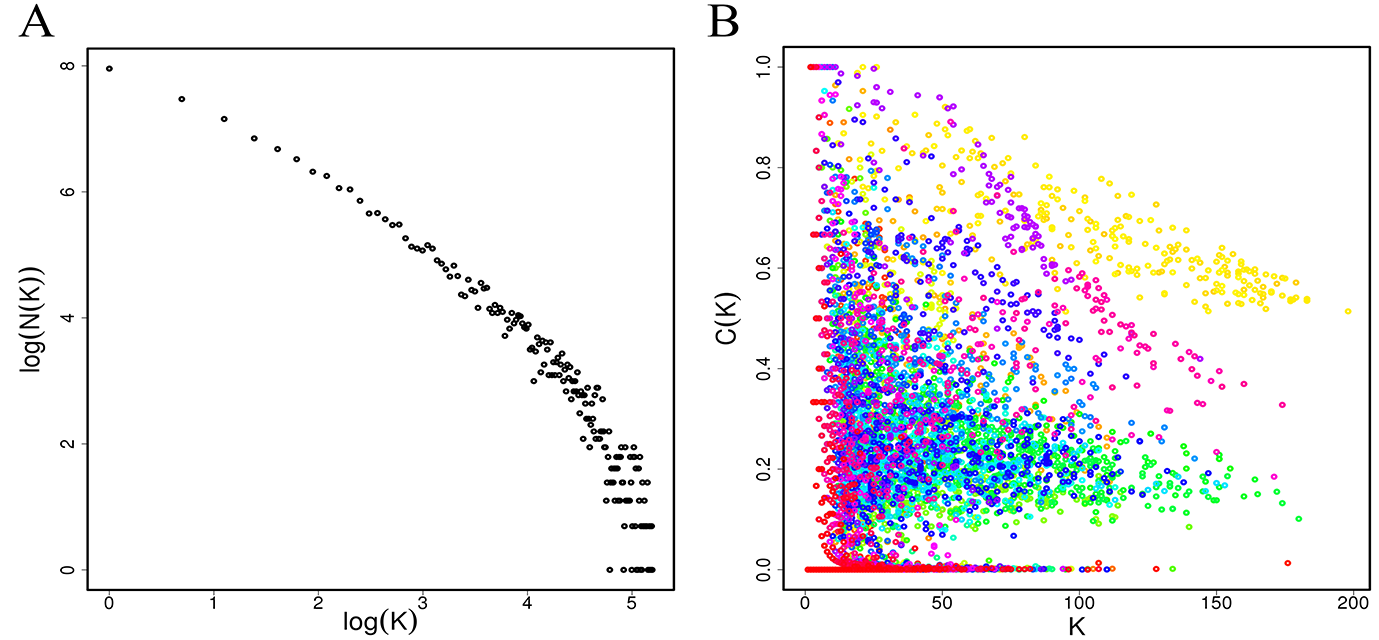


**S9 Fig** Network topological analysis. A) The degree distribution, K denotes degree and N(K) represented the number of nodes with degree K*.* B) The scatter plots of degree K versus the clustering coefficient C(K) of any node having K edges. Data points with different colors denoted the genes located in different co-expression modules obtained by Markov CLuster (MCL)
