# Supplementary material for: NetMiner-an ensemble pipeline for building genome-wide and high-quality gene co-expression network using massive-scale RNA-seq samples
Source: PLoS One. 2018 Feb 9;13(2):e0192613. doi: 10.1371/journal.pone.0192613 (PMC5806890; doi:10.1371/journal.pone.0192613)
Supplement: S10 Fig — (DOC) [file pone.0192613.s015.doc]

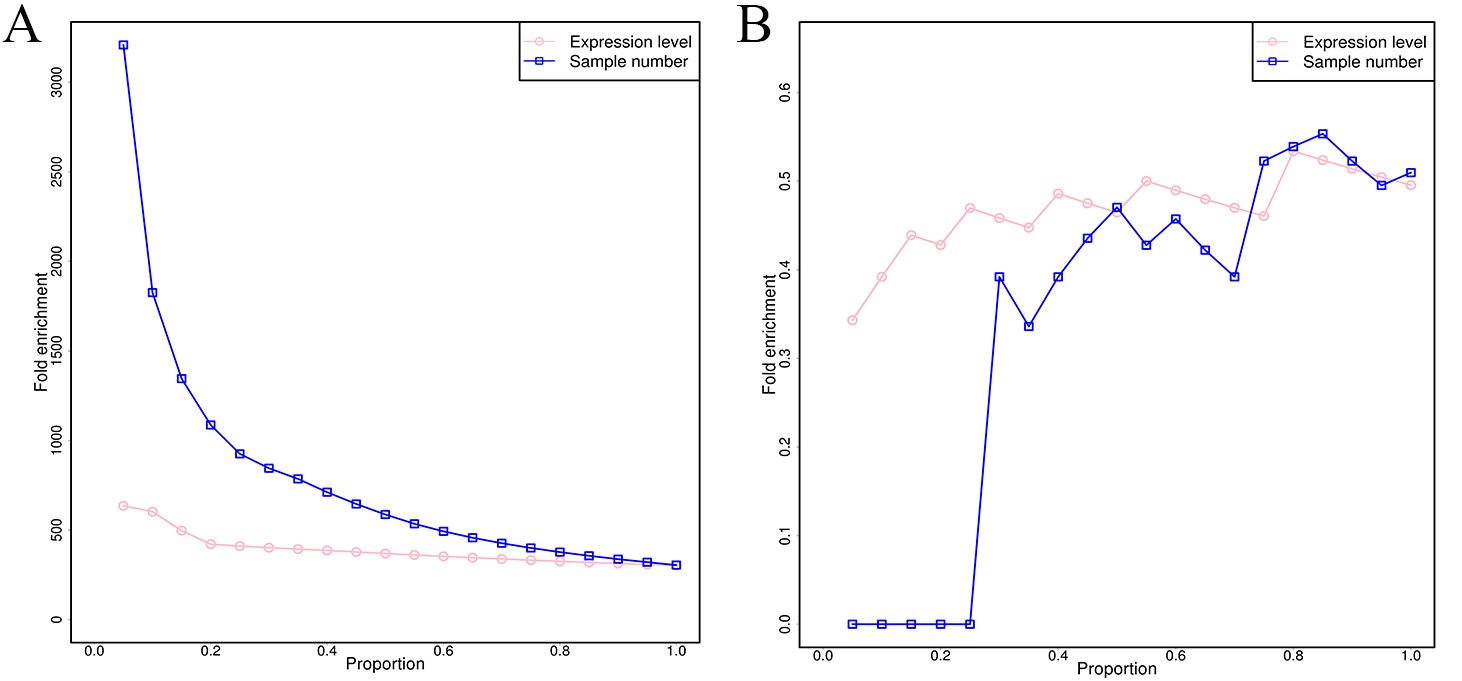


**S10 Fig** The enrichment fold curve of co-expression links based on expression abundance ranking and expression sample number ranking. The expression abundance of a co-expression link, connecting two gene A and B, was defined as the total number samples which simply plus the number of gene A expressed samples and the number of gene B expressed samples. The expression sample number of a co-expression link, connecting two gene A and B, was defined as the expression abundance summation of gene A and gene B. A) Standard positive links. B) Standard negative links
