# Supplementary material for: NetMiner-an ensemble pipeline for building genome-wide and high-quality gene co-expression network using massive-scale RNA-seq samples
Source: PLoS One. 2018 Feb 9;13(2):e0192613. doi: 10.1371/journal.pone.0192613 (PMC5806890; doi:10.1371/journal.pone.0192613)
Supplement: S11 Fig — (DOC) [file pone.0192613.s016.doc]

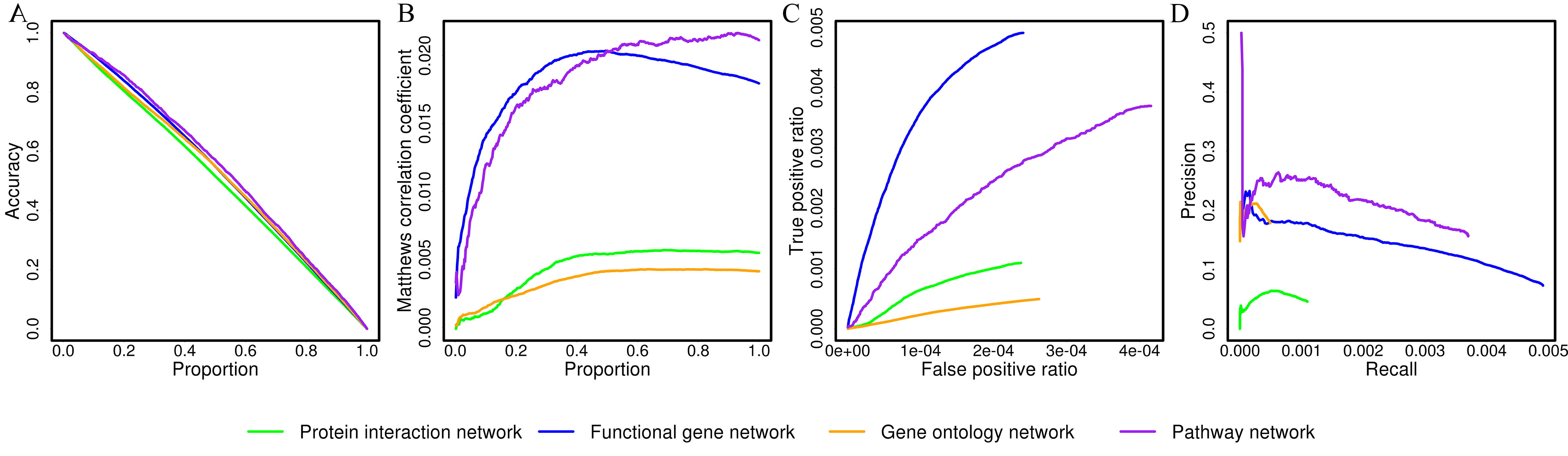


**S11 Fig** Assessment of the overlap and coherence between reconstructed rice RNA-seq-based gene co-expression networks and reference rice networks. A) Accuracies at different proportions of co-expression links. B) Matthews Correlation Coefficients (MCCs) at different proportions of co-expression links. C) Receiver Operating Characteristic curves. D) Precision-Recall curves. The accuracies were calculated using$\text{(TP+TN)/(TP+TN+FP+FN)}$, and were normalized to interval ranging from 0 to 1. The MCC was calculated as $\text{(TP}\times\text{TN-FN}\times\text{FP)/}\sqrt{\text{(TN+FN)}\times\text{(TP+FN)}\times\text{(TN+FP)}\times\text{(TP+FP)}}$. Receiver Operating Characteristics (ROC) curve was plotted by changing the threshold and plotting the sensitivity versus the 1-specificity and then calculated the value of AUC. Similarly, we plotted Precision-Recall (PR) curve by altering the threshold and plotting the Precision versus the Recall. Sensitivity (Recall) was calculated as$\text{TP}/\text{(TP+FN})$. 1-specificity was calculated as $\text{TN}/\text{(TN+FP})$. Precision was calculated as$\text{TP}/\text{(TP+FP)}$. Where TP = True Positives. FP = False Positives. TN = True Negatives. FN = False Negatives
