# Supplementary material for: NetMiner-an ensemble pipeline for building genome-wide and high-quality gene co-expression network using massive-scale RNA-seq samples
Source: PLoS One. 2018 Feb 9;13(2):e0192613. doi: 10.1371/journal.pone.0192613 (PMC5806890; doi:10.1371/journal.pone.0192613)
Supplement: S12 Fig — (DOC) [file pone.0192613.s017.doc]

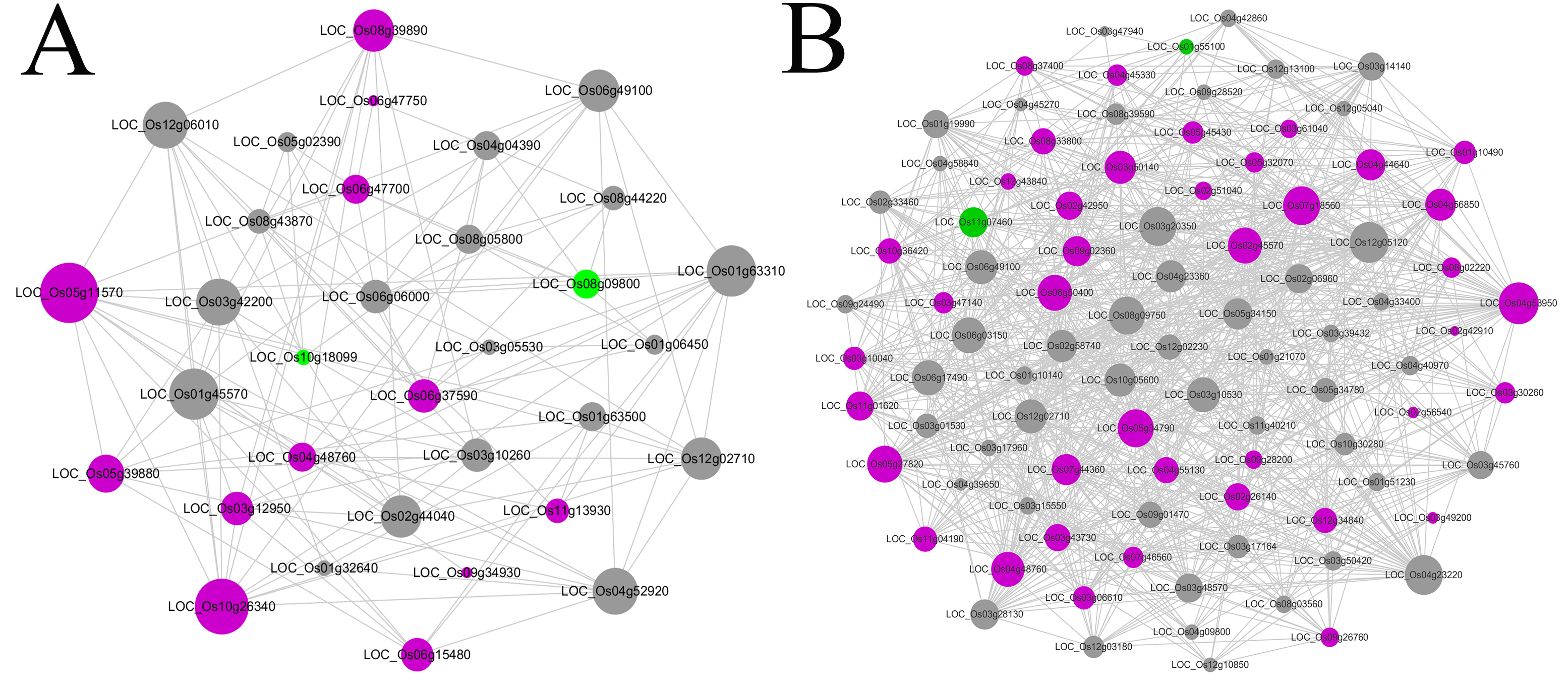


**S12 Fig** Subnetworks derived from the miRNA-guide approach. A) miRNA156. B) miRNA396. Pink nodes represented the genes involved in cell division, cell proliferation, cell differentiation and development process; green nodes indicated the transcription factors and gray nodes denoted that the genes have not known functions or annotated with the irrelevant functions. The size of node was proportional to the number of connected genes. We only selected the genes with *p*-value smaller than 0.05 and the number of the enriched targets larger than 10 for demonstration
