# Supplementary material for: NetMiner-an ensemble pipeline for building genome-wide and high-quality gene co-expression network using massive-scale RNA-seq samples
Source: PLoS One. 2018 Feb 9;13(2):e0192613. doi: 10.1371/journal.pone.0192613 (PMC5806890; doi:10.1371/journal.pone.0192613)
Supplement: S13 Fig — (DOC) [file pone.0192613.s018.doc]

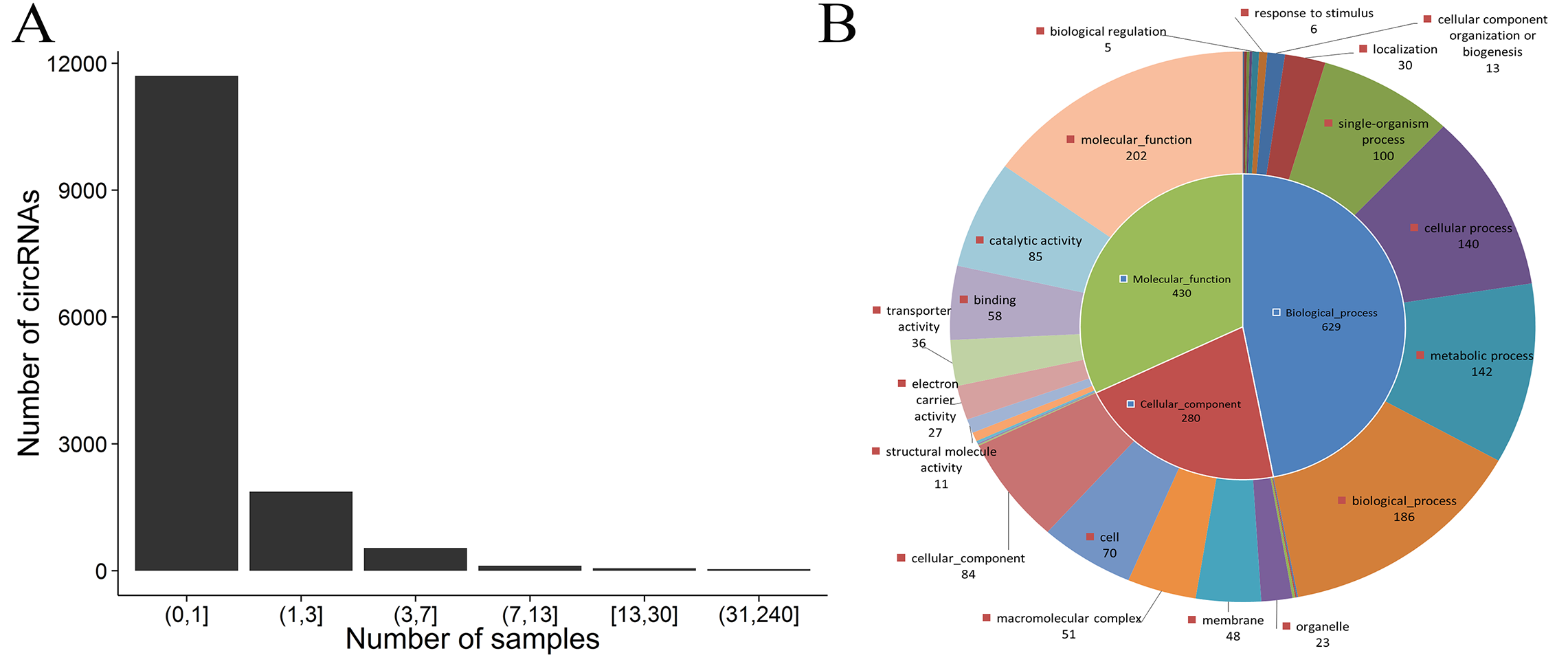


**S13 Fig** The statistic results of circRNAs. A, The distribution of the number of circRNA detected in different intervals of sample number. B, The distribution of predicted biological functions for circRNAs
