# Supplementary material for: NetMiner-an ensemble pipeline for building genome-wide and high-quality gene co-expression network using massive-scale RNA-seq samples
Source: PLoS One. 2018 Feb 9;13(2):e0192613. doi: 10.1371/journal.pone.0192613 (PMC5806890; doi:10.1371/journal.pone.0192613)
Supplement: S1 Table — (DOC) [file pone.0192613.s019.doc]

**S1 Table** The representative functional categories in which the tissue-specific up-expressed genes were enriched

| **Tissue** | **Term/Pathway ID** | **Term/Pathway name** | ***q*-value (Fisher’s exact test)** | |
| --- | --- | --- | --- | --- |
| Callus | PWY-5784 | IAA conjugate biosynthesis II | 1.55E-3 |  |
| Panicle | GO:0009908 | flower development | 1.24E-3 |  |
| Pollen | GO:0006261 | DNA-dependent DNA replication | 1.65E-3 |  |
| Pollen | map03440 | homologous recombination | 8.82E-3 |  |
| Pollen | GO:0007165 | signal transduction | 5.87E-4 |  |
| Pollen | GO:0006281 | DNA repair | 3.65E-2 |  |
| Root | GO:0006952 | defense response | 1.68E-6 |  |
| Root | GO:0030145 | manganese ion binding | 2.28E-7 |  |
| Root | GO:0005507 | copper ion binding | 6.34E-6 |  |
| Shoot | GO:0015979 | Photosynthesis | 1.22E-30 |  |
| Shoot | GO:0009579 | Thylakoid | 5.99E-15 |  |
| Shoot | GO:0009507 | Chloroplast | 6.96E-9 |  |
| Shoot | PWY-181 | photorespiration | 2.89E-2 |  |
