# Supplementary material for: NetMiner-an ensemble pipeline for building genome-wide and high-quality gene co-expression network using massive-scale RNA-seq samples
Source: PLoS One. 2018 Feb 9;13(2):e0192613. doi: 10.1371/journal.pone.0192613 (PMC5806890; doi:10.1371/journal.pone.0192613)
Supplement: S2 Table — (DOC) [file pone.0192613.s020.doc]

**S2 Table The statistic table of agronomic traits whose genes were highly connected in our network**

| **Agronomic trait** | **Total number of genes** | **Number of expressed genes** | **Number of genes contained in network** | **# of all co-expression links** | **# of links between agronomic trait genes** | **Enrichment fold** | ***p*-value (Fisher’s exact test)** | **Percentage of permutation test times ^b^** |
| --- | --- | --- | --- | --- | --- | --- | --- | --- |
| Source activity ^a^ | 84 | 84 | 61 | 2233 | 91 | 47.81 | 3.96E-117 | 0 |
| Culm leaf ^a^ | 169 | 169 | 101 | 2353 | 28 | 5.33 | 3.03E-12 | 3 |
| Panicle flower ^a^ | 115 | 115 | 67 | 1122 | 18 | 7.83 | 5.57E-11 | 7 |
| Drought tolerance ^a^ | 102 | 100 | 67 | 1470 | 36 | 15.66 | 2.45E-30 | 0 |
| Salinity tolerance ^a^ | 95 | 95 | 62 | 1360 | 23 | 11.70 | 3.09E-17 | 0 |
| Other soil stress tolerance ^a^ | 61 | 61 | 44 | 850 | 17 | 15.79 | 3.24E-15 | 1 |
| Eating quality ^a^ | 52 | 51 | 24 | 348 | 9 | 31.35 | 2.52E-11 | 1 |
| Cold tolerance ^a^ | 41 | 41 | 25 | 751 | 9 | 28.85 | 5.27E-11 | 2 |
| Lodging resistance ^a^ | 16 | 16 | 10 | 122 | 7 | 149.57 | 5.82E-14 | 0 |

^a^ represented the agronomic traits extracted from Q-TARO database and literatures; ^b^ denoted the percentage of test times with the number of co-expression links larger than the number of links between agronomic trait genes in 1000 permutation. Permutation test was carried out by randomly selecting the same number genes of given agronomic trait from all agronomic trait related genes.
